# RNA SEQ Analysis Indicates that the AE3 Cl^−^/HCO_3_^−^ Exchanger Contributes to Active Transport-Mediated CO_2_ Disposal in Heart

**DOI:** 10.1038/s41598-017-07585-y

**Published:** 2017-08-04

**Authors:** Kanimozhi Vairamani, Hong-Sheng Wang, Mario Medvedovic, John N. Lorenz, Gary E. Shull

**Affiliations:** 1 Department of Molecular Genetics, Biochemistry and Microbiology, University of Cincinnati College of Medicine, Cincinnati, Ohio 45267 USA; 2 Department of Pharmacology and Cell Biophysics, University of Cincinnati College of Medicine, Cincinnati, Ohio 45267 USA; 3 Department of Environmental Health, University of Cincinnati College of Medicine, Cincinnati, Ohio 45267 USA; 4 Department of Cellular and Molecular Physiology, University of Cincinnati College of Medicine, Cincinnati, Ohio 45267 USA

## Abstract

Loss of the AE3 Cl^−^/HCO_3_^−^ exchanger (*Slc4a3*) in mice causes an impaired cardiac force-frequency response and heart failure under some conditions but the mechanisms are not known. To better understand the functions of AE3, we performed RNA Seq analysis of AE3-null and wild-type mouse hearts and evaluated the data with respect to three hypotheses (CO_2_ disposal, facilitation of Na^+^-loading, and recovery from an alkaline load) that have been proposed for its physiological functions. Gene Ontology and PubMatrix analyses of differentially expressed genes revealed a hypoxia response and changes in vasodilation and angiogenesis genes that strongly support the CO_2_ disposal hypothesis. Differential expression of energy metabolism genes, which indicated increased glucose utilization and decreased fatty acid utilization, were consistent with adaptive responses to perturbations of O_2_/CO_2_ balance in AE3-null myocytes. Given that the myocardium is an obligate aerobic tissue and consumes large amounts of O_2_, the data suggest that loss of AE3, which has the potential to extrude CO_2_ in the form of HCO_3_^−^, impairs O_2_/CO_2_ balance in cardiac myocytes. These results support a model in which the AE3 Cl^−^/HCO_3_^−^ exchanger, coupled with parallel Cl^−^ and H^+^-extrusion mechanisms and extracellular carbonic anhydrase, is responsible for active transport-mediated disposal of CO_2_.

## Introduction

Anion exchanger isoform 3 (AE3; gene symbol *Slc4a3*), the most abundant Cl^−^/HCO_3_^−^ exchanger in cardiac muscle^1^, mediates electroneutral extrusion of HCO_3_^−^ in exchange for inward transport of Cl^−^. Although its transport function is well understood and it is the major HCO_3_^−^ extrusion mechanism in cardiac myocytes^2^, its physiological function is unclear. Mice lacking AE3 appear healthy and exhibit normal contractility under some conditions^3^; however, they have an impaired cardiac force-frequency response^4^ and develop rapid decompensation and heart failure on a hypertrophic cardiomyopathy background^5^. Proposed physiological functions for AE3 include operating in concert with Na^+^/H^+^ exchanger isoform 1 (NHE1) to facilitate Na^+^-loading, with subsequent effects on Ca^2+^-loading^2, 6–8^, and mediating recovery of intracellular pH (pH_i_) from an alkaline load^2, 9^. Although these functions are possible, the lack of an effect of AE3 ablation on hypertrophy *in vivo* ^5^ or Ca^2+^-transients in isolated myocytes^4^ and the high metabolic acid load *in vivo*, particularly from CO_2_ hydration, suggest that these are not its major functions.

The RNA Seq data reported here provide strong support for a third hypothesis, originally proposed for retinal and neuronal cells^10–12^, that AE3-mediated HCO_3_^−^ extrusion contributes to CO_2_ disposal. This hypothesis is consistent with data showing that intracellular carbonic anhydrase (CA) facilitates CO_2_ venting from cardiomyocyte mitochondria by generating HCO_3_^−^ and H^+^, and that this conversion is necessary to avoid inhibition of oxidative phosphorylation by waste CO_2_^13^. These findings suggest the need to dispose of the CO_2_ hydration products (H^+^  + HCO_3_^−^) rather than simply CO_2_ itself. Furthermore, extracellular CA is associated with AE3^12, 14^, indicating that HCO_3_^−^ extruded by AE3 is combined with H^+^ extruded via some other mechanism to form CO_2_ on the extracellular surface. In fact, the association of AE3 and extracellular CA has been cited previously as supporting the CO_2_ disposal hypothesis^12^. A direct correlate of the hypothesis that AE3 contributes to CO_2_ disposal is that this mechanism would require energetically-efficient H^+^ extrusion and overall charge balance, which cannot be provided by the known acid-extrusion mechanisms in myocytes. However, data in publically available expression databases shows that the HVCN1 voltage-sensitive H^+^ channel, which would provide both energetically-efficient H^+^-extrusion and charge balance, is expressed in all mammalian tissues, including heart. These observations and the current RNA Seq data suggest that the combined activities of AE3 and HVCN1, in combination with Cl^−^ recycling and extracellular CA activity, contribute to transport-mediated CO_2_ disposal on a beat-to-beat basis.

## Results

### RNA Seq analysis of wild-type (WT) and AE3-null hearts

RNA Seq analysis was performed to obtain differential expression data that might support or negate one or more of the three major hypotheses that have been proposed for the physiological functions of AE3. In order of their perceived strengths (See Supplementary Results and Discussion), these are: (i) CO_2_ disposal, (ii) stimulation of Na^+^- and Ca^2+^-loading, and (iii) recovery from an intracellular alkaline load.

Gene Ontology analysis^15^ of the entire data set (Supplementary Table S1) and PubMatrix literature analyses^16^ of genes with a False Discovery Rate (FDR) <0.05 revealed differential expression patterns involving hypoxia, angiogenesis, energy metabolism, and cardiac electrical and myofibrillar functions (Table 1 and Supplementary Tables S2–S7). As explained below, the observed expression patterns provide strong support for the CO_2_ disposal hypothesis and limited support for the Na^+^-loading hypothesis.

**Table 1:** Significantly enriched Gene Ontology (GO) categories.

GO Category	P-value	Enrichment	(N, B, n, b)
**Hypoxia/Angiogenesis/Vasodilation**			
GO:0001525 Angiogenesis	8.08E-12	3.57	(21238,231,979,38)
GO:0001666 Response to hypoxia	7.5E-05	2.71	(21238,152,979,19)
GO:0019229 Regulation of vasoconstriction	6.51E-04	3.39	(21238,64,979,10)
GO:0042312 Regulation of vasodilation	7.86E-04	3.96	(21238,44,979,8)
**Lipid/Carbohydrate metabolism**			
GO:0006109 Regulation of carbohydrate metabolic process	2.74E-07	8.79	(21239,151,176,11)
GO:0010906 Regulation of glucose metabolic process	6.16E-05	4.6	(21257,99,607,13)
GO:0008286 Insulin receptor signaling pathway	8.09E-05	6.52	(21257,49,599,9)
GO:0019216 Regulation of lipid metabolic process	1.11E-04	2.57	(21257,230,863,24)
GO:0032868 Response to Insulin	4.36E-04	3.84	(21257,120,599,13)
GO:0006110 Regulation of Glycolytic Process	3.48E-04	19.35	(21257,26,169,4)
**Cardiac conduction/Transporters/Channels**			
GO:0008016 Regulation of heart contraction	3.76E-10	4.22	(21228,132,990,26)
GO:0061337 Cardiac conduction	2.22E-08	8.42	(21228,28,990,11)
GO:0002027 Regulation of heart rate	1.55E-08	5.21	(21228,70,990,17)
GO:0006811 Ion transport	3.56E-06	1.67	(21239,1054,990,82)
GO:0006813 Potassium ion transport	2.72E-05	2.82	(21239,152,990,20)
GO:0086001 Cardiac muscle cell action potential	1.89E-07	7.95	(21239,27,990,10)
GO:0051899 Membrane depolarization	1.59E-04	3.09	(21228,97,990,14)
GO:0086015 SA node cell action potential	3.9E-04	16.09	(21239,4,990,3)
GO:0086069 Bundle of His cell to Purkinje myocyte communication	4.9E-04	9.53	(21239,9,990,4)
GO:0086067 AV node cell to bundle of His cell communication	9.41E-04	12.87	(21239,5,990,3)
GO:0006816 Calcium ion transport	9.84E-04	2.17	(21239,198,990,20)
**Sarcomere/Z-disc/Cytoskeleton**			
GO:0044449 Contractile fiber part	2.93E-14	4.57	(21238,166,979,35)
GO:0032432 Actin filament bundle	3.06E-11	6.54	(21238,63,979,19)
GO:0042641 Actomyosin	4.97E-09	5.59	(21238,66,979,17)
GO:0030018 Z disc	8.18E-09	4.62	(21238,94,979,20)
GO:0030315 T-tubule	2.19E-07	5.76	(21238,49,979,13)
GO:0014704 Intercalated disc	3.71E-05	4.72	(21238,46,979,10)

GO categories were identified using the GOrilla program^15^ and grouped by related functions as described in Methods and Supplementary Information. The degree of enrichment = (b/n)/(B/N), with (N, B, n, b) defined as follows: N is the total number of genes, B is the total number of genes associated with a specific GO term, n is the number of genes in the target set, b is the number of genes in the intersection. For more complete information on GO categories and how to access specific gene lists in Excel Files, see legends for Supplementary Tables 2–7.

### Expression changes indicate mild hypoxia and vasodilation

**Figure 1:**
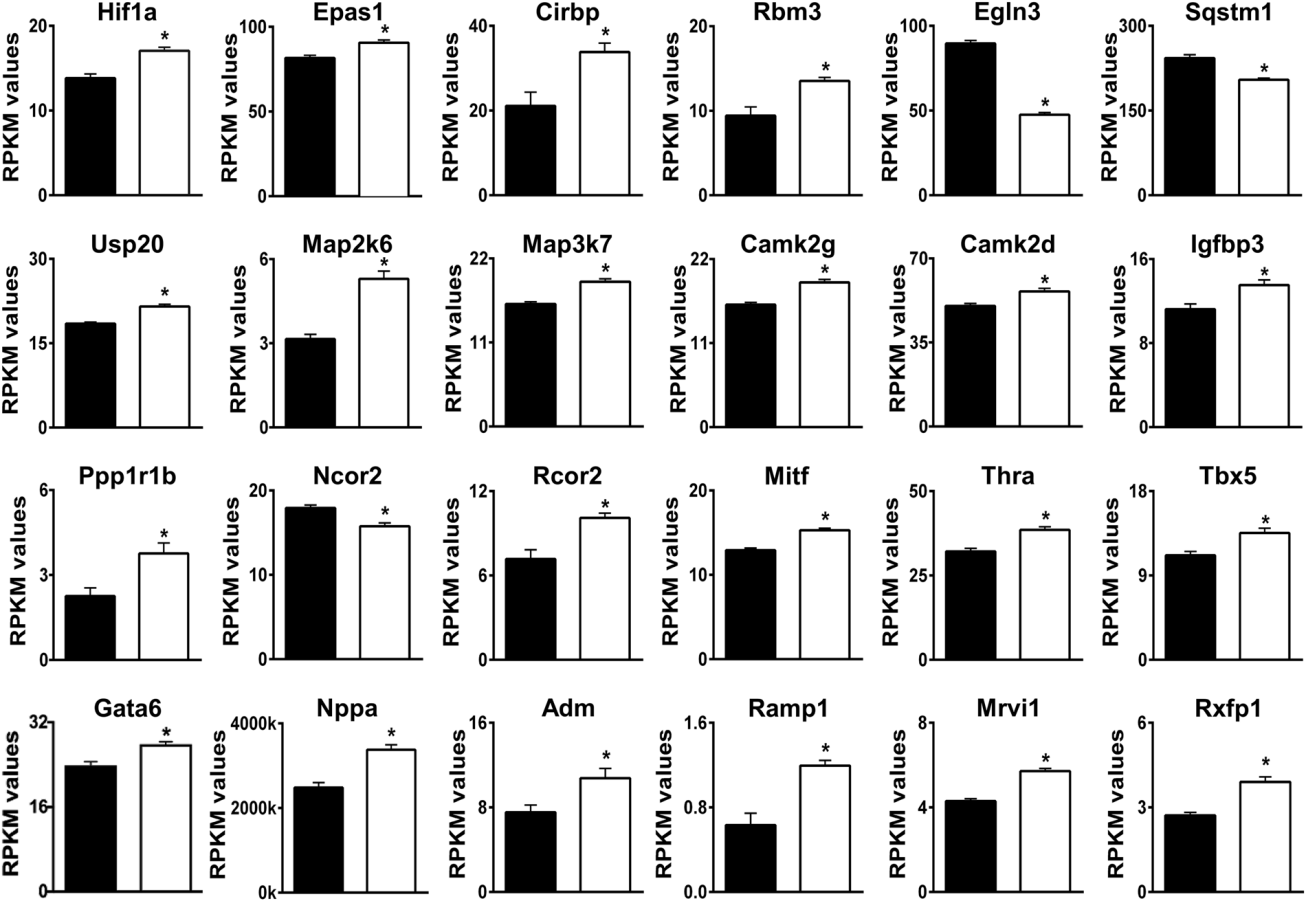
Differential expression of genes involved in hypoxia responses and vasodilation. Relevant genes were identified by Gene Ontology analyses and/or by PubMatrix analyses of genes with an FDR < 0.05. RPKM (Reads Per Kilobase of transcript per Million mapped reads) values for WT (black bars) and AE3-null (white bars) hearts are shown. The genes shown are a subset of 143 genes in Supplementary Table S2 and in a subset of 158 signaling genes in Supplementary Table S3; values are means ± SE; n = 4 for each genotype; *p ≤ 0.01 vs WT controls.

Changes indicating a hypoxia response in AE3-null hearts (Fig. 1) included small increases in mRNAs for the transcription factors **Hif1a** and **Epas1** (Hif2a), which play a central role in hypoxia responses^17–19^, and larger changes for the RNA binding proteins **Cirbp** and **Rbm3**, which mediate Hif1a-independent hypoxia responses^20^. Also included were genes involved in direct regulation of hypoxia responses by a variety of mechanisms. **Egln3** (prolyl hydroxylase 3), one of the most significantly down-regulated genes, contributes to O_2_-mediated degradation of Hif1a and other substrates^21^. **Egln3** is normally up-regulated in hypoxia, an apparent discrepancy; however, it is down-regulated in high altitude hypoxia^22^, suggesting that under mild hypoxia, in which O_2_ is available at sufficient levels to stimulate enzyme activity, reduced Egln3 expression would limit prolyl hydroxylase activity^22^. **Sqstm1** (Sequestosome 1) is down-regulated by hypoxia and regulates metabolism and degradation of proteins, including Egln3^23^. Up-regulation of **Usp20**, a deubiquitinase that acts on Hif1a and other proteins^24^, should reduce degradation of Hif1a.

Among major signaling proteins, **Map2k6**, which is involved in activation of Hif1a during hypoxia^25^, and **Map3k7**, which is required for hypoxia-induced NF-kB activity^26^, were up-regulated. **Camk2g** and **Camk2d** were up-regulated; both genes are in the hypoxia response GO category and it is known that Ca^2+^/calmodulin-dependent protein kinase II contributes to cardioprotection after intermittent hypoxia^27^. **Igfbp3** (insulin-like growth factor-binding protein-3), a Hif1a target involved in many signaling pathways^28^, was increased. **Ppp1r1b**, an inhibitory subunit of protein phosphatase 1 that is induced by hypoxia^29^, was also up-regulated.

**Ncor2**, a transcriptional corepressor that inhibits Hif1a-mediated transcription^30^ was down-regulated and additional hypoxia-related transcription factors were affected. These include increased expression of **Rcor2**, a transcriptional corepressor that is a Hif1a target and up-regulated by hypoxia^31^, and both **Mitf** (microphthalmia associated transcription factor) and **Thra** (thyroid hormone receptor α), which are known to up-regulate Hif1a mRNA^32–34^. Also included was **Tbx5**, a major cardiac transcription factor that is a Hif1a target^35^, is up-regulated by hypoxia^35^, and works synergistically with **Gata6**, a member of the hypoxia response GO category, to induce atrial natriuretic factor^36^.

Changes indicating increased vasodilation (Fig. 1) included up-regulation of **Nppa** (ANF, atrial natriuretic factor) and **Adm** (adrenomedullin), which are induced by hypoxia^37, 38^. Both proteins are secreted from myocytes and mediate vasodilation^39^. Also included were up-regulation of **Ramp1**, which can form part of an adrenomedulin receptor^40^, and **Mrvi1** (IRAG) and **Rxfp1**, which mediate smooth muscle relaxation in response to ANF^41^ and relaxin^42^, respectively. Down-regulation of **Ednrb** (endothelin receptor type B; 0.84-fold; not shown) could also contribute to vasodilation^43^. These expression changes would be expected to increase blood flow and O_2_ delivery to stromal tissue in heart, thus providing some compensation for mild hypoxia occurring in myocytes as a result of impaired CO_2_ disposal.

### Expression changes indicate reduced angiogenesis

**Figure 2:**
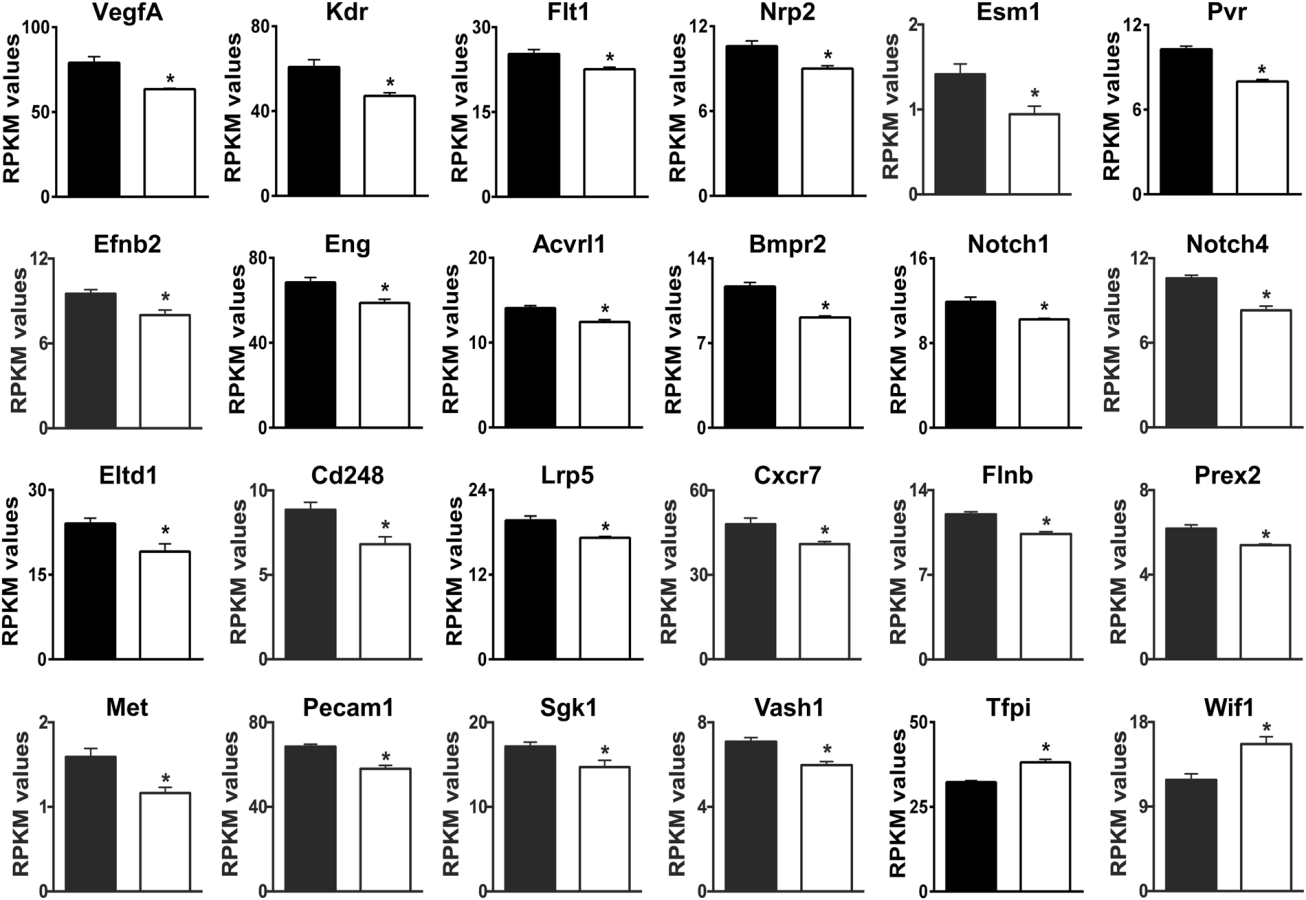
Differential expression of genes involved in angiogenesis. Relevant genes were identified by Gene Ontology analyses and/or by PubMatrix analyses of genes with an FDR < 0.05. RPKM values for WT (black bars) and AE3-null (white bars) hearts are shown. The genes shown are a subset of 143 genes in Supplementary Table S2; values are means ± SE; n = 4 for each genotype; *p ≤ 0.01 vs WT controls.

Changes in genes that have clear vascular functions and affect angiogenesis (Fig. 2) included down-regulation of **VegfA**, which plays a central role in angiogenesis, along with its receptors **Kdr** (VEGFR2) and **Flt1** (VEGFR1), its coreceptor **Nrp2** ^44, 45^, and **Esm1** (endocan), **Pvr** (Necl-5), and **Efnb2** (Ephrin B2), all three of which regulate of VEGFA/VEGFR2-driven angiogenesis^46–49^. **Eng** (endoglin), **Acvrl1** (ALK1), and **Bmpr2**, which interact and serve as receptors for bone morphogenetic proteins during angiogenesis, were also down-regulated^50^, along with many other proteins with established roles in angiogenesis. These include: **Notch1**, **Notch4**, and the Notch ligand **Dll4** (0.81-fold, not shown)^51, 52^; **Eltd1**, a G-protein coupled receptor that regulates sprouting angiogenesis and interacts with the Dll4-Notch pathway^53^; and **CD248** (endosialin), which also regulates sprouting angiogenesis and acts via a pathway involving platelet-derived growth factor B (**Pdgfb**, 0.89-fold, not shown)^54^. Other down-regulated genes and some of their pro-angiogenesis functions are: **Lrp5**, regulation of angiogenesis as part of the Wnt receptor complex^55^; **Cxcr7**, a chemokine receptor that is regulated by VEGFA/VEGFR2 signaling and regulates endothelial progenitor cell tube formation^56, 57^; **Flnb** (Filamin B), endothelial cell migration in response to VEGF stimulation^58^; **Prex2** (a guanine nucleotide exchange factor)^59^ and **Met** (receptor for hepatocyte growth factor)^60^, both involved in endothelial cell migration via signaling mechanisms involving Rac1; **Pecam1**, modulates endothelial cell-cell and cell-matrix interactions^61^; **Sgk1**, endothelial cell migration and tube formation^62^; and **Vash1**, negative feedback regulation of angiogenesis after transcriptional induction by VEGFA and other pro-angiogenic stimuli^63^. **Tfpi** and **Wif1**, which were up-regulated, both serve as inhibitors of angiogenesis^64, 65^. In light of the apparent hypoxia response, the direction of changes in angiogenesis genes might seem paradoxical. However, this is consistent with mild hypoxia in AE3-null myocytes due to an O_2_/CO_2_ imbalance, with increased oxygenation of stromal tissue leading to reduced angiogenesis.

### Expression changes indicate altered energy metabolism with greater reliance on glucose and lesser reliance on fatty acids for energy metabolism

**Figure 3:**
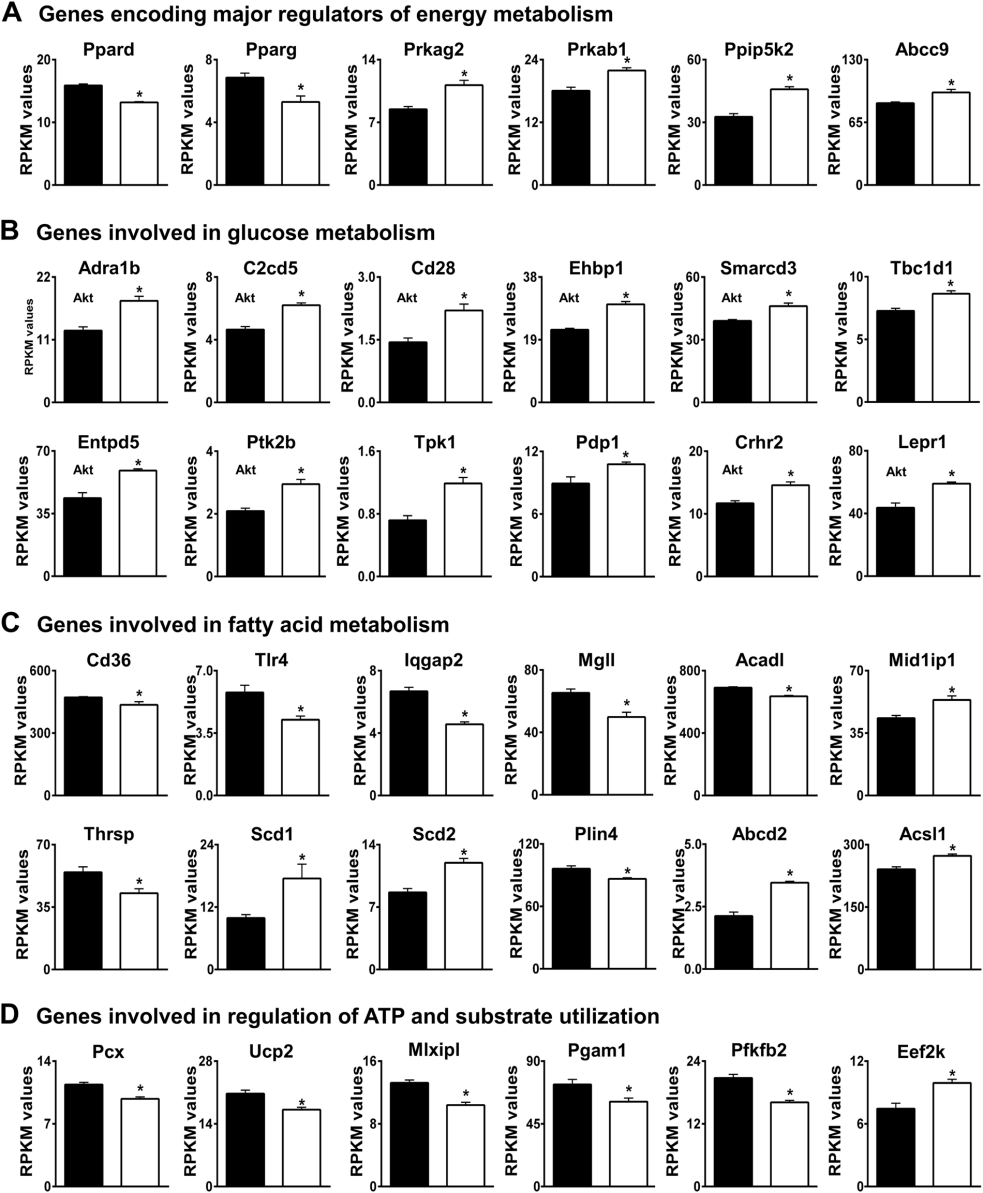
Differential expression of genes involved in energy metabolism. Genes encoding proteins that function in (**A**) regulation of energy metabolism, (**B**) glucose metabolism, (**C**) fatty acid metabolism, and (**D**) regulation of ATP and substrate utilization were identified by Gene Ontology analyses and/or by PubMatrix analyses of genes with an FDR < 0.05. RPKM values for WT (black bars) and AE3-null (white bars) hearts are shown. Genes encoding proteins that are regulated by Akt are indicated (Akt). The genes shown are a subset of 142 genes in Supplementary Table S4; values are means ± SE; n = 4 for each genotype; *p ≤ 0.01 vs WT controls.

CO_2_ venting from mitochondria is facilitated by intracellular CA-mediated hydration of CO_2_ to HCO_3_^−^ and H^+^ and a block in this conversion has been shown to inhibit oxidative phosphorylation^13^. Thus, if AE3-null myocytes have impaired CO_2_ disposal, one would predict changes in energy metabolism and substrate utilization to make the heart more efficient with respect to O_2_ utilization. Glycolysis and glucose oxidation are more efficient for ATP generation than fatty acid oxidation, and a reduction in the use of ATP and substrates for biosynthesis would allow greater utilization of ATP for contraction. Expression changes in energy metabolism GO categories were highly significant (Table 1), and when the directions of changes were examined, they indicated increased glucose metabolism, reduced fatty acid metabolism, and reduced biosynthesis (Fig. 3).

Changes in genes encoding major regulators of energy metabolism (Fig. 3A) included down-regulation of the transcription factors **Ppard** (a member of the hypoxia response GO category) and **Pparg**. The expression of both Ppard and Pparg are reduced by hypoxia^66, 67^, consistent with a role for AE3 in maintenance of O_2_/CO_2_ balance. **Prkag2** and **Prkab1**, regulatory subunits of AMPK, which plays a major role in energy sensing, energy metabolism^68^, and response to hypoxia^69^, were up-regulated. **Ppip5k2** (diphosphoinositol pentakisphosphate kinase 2, 1.38-fold increase, not shown) regulates inositol pyrophosphate metabolism and is an AMPK-independent energy sensor and regulator^70^. The hypoxia-responsive ATP-sensitive K^+^ channels, **Abcc8** (Sur1) and **Abcc9** (Sur2), were up-regulated. Both channels interact with Kcnj11 (Kir6.2, not changed), with Abcc8 the predominant form in atria and Abcc9 the predominant form in ventricles^71^. Abcc9 protects the heart against ischemia and both channels serve as metabolic sensors, couple energy metabolism and membrane excitability, play major roles in carbohydrate metabolism, and are induced by hypoxia^72, 73^.

Expression of genes for proteins that stimulate glycolysis, glucose uptake, and glucose oxidation were increased. These include **Hif1a** and **Thra** (Fig. 1), which play major roles in glucose metabolism^74, 75^. Changes in additional genes involved in glucose metabolism are shown in Fig. 3B. The α1-adrenergic receptor (**Adra1b**) is cardioprotective during myocardial infarction and ischemia in part because of enhanced glucose metabolism^74, 76^. Many of the encoded proteins are affected by activation of Akt (indicated in Fig. 3B). This occurs via phosphorylation of Ser473, which was significantly increased in AE3-null hearts subjected to atrial pacing^4^. For example, **C2cd5** contributes to insertion of GLUT4 into the plasma membrane^77^, **Cd28** stimulates glucose uptake and glycolysis^78^, and **Ehbp1** is involved in insulin-regulated GLUT4 recycling and glucose transport^79^, all in response to Akt activation. **Smarcd3** is a transcriptional cofactor that drives glycolytic metabolism through a mechanism involving Akt^80^. **Entpd5**, a UDPase, is involved in Akt responses and has been shown to increase the catabolic efficiency of aerobic glycolysis in tumor cells^81^. **Ptk2b** (Pyk2 tyrosine kinase, focal adhesion kinase 2) mediates insulin-independent insertion of GLUT4 into the plasma membrane^82^ and contributes to α1-adrenergic receptor-mediated activation of Akt^83^. **Tpk1** (thiamine pyrophosphokinase), generates thiamine pyrophosphate, a cofactor needed for oxidative decarboxylation of pyruvate and other substrates in mitochondria^84^. Up-regulation of **Pdp1** (pyruvate dehydrogenase phosphatase 1) would favor dephosphorylation and activation of pyruvate dehydrogenase, which would enhance pyruvate (glucose) oxidation^85^. Activation of **Crhr2**, the urocortin 2 receptor, causes increased AMPK activation, glucose uptake, and phosphorylation of acetyl-CoA carboxylase (which inhibits fatty acid biosynthesis) in cardiomyocytes^86^; cardioprotective effects of Crhr2 activation during ischemic injury include activation of phosphatidylinositol 3-kinase/Akt signaling^87^. Gene knockout studies showed that loss of **Tbc1d1** impairs insulin-stimulated glucose uptake and increases fatty acid oxidation^88^, so up-regulation of Tbc1d1 should favor a switch to glucose metabolism.

Expression of the leptin receptor (**Lepr**), which is induced by hypoxia^89^ and affects both glucose and lipid metabolism, was up-regulated (Fig. 3C). In isolated hearts, treatment with leptin stimulates fatty acid oxidation, reduces cardiac efficiency, and has no effect on glucose oxidation^90^ so one could question a role for its receptor in glucose metabolism; however, hearts of diabetic mice lacking a functional long form of Lepr (ObRb), which might be expected to show the opposite effect, exhibit an increase in palmitate oxidation and a decrease in glycolysis and glucose oxidation^91^. Also, after myocardial infarction, Lepr was upregulated and contributed to a shift from fatty acid to glucose metabolism in a process involving phosphatidylinositol 3-kinase/Akt signaling^92^. The latter study raises the possibility that up-regulation of Lepr mRNA in AE3-null hearts, which includes increased expression of the long form of Lepr (Supplementary Fig. S1), may affect glucose metabolism.

Down-regulation of Pparg and Ppard (Fig. 3A) is consistent with reduced fatty acid oxidation, as it has been shown that cardiac-specific ablation of Pparg^93^ or Ppard^94^ causes a reduction in fatty acid oxidation. Also, hypoxia-induced microRNA-mediated repression of Ppard facilitates a reduction in fatty acid metabolism and an increase in glucose metabolism in the failing heart^66^. Reduced utilization of fatty acids for energy metabolism is also suggested by reduced expression of the following genes (Fig. 3C): **Cd36**, which mediates fatty acid uptake across the plasma membrane^95^; **Tlr4**, which interacts with Cd36 and stimulates fatty acid uptake^96^; **Iqgap2**, which also interacts with Cd36 and serves a signaling pathway that stimulates fatty acid uptake and processing^97^; **Mgll**, which hydrolyzes monoglycerides to produce fatty acids and glycerol for energy metabolism and biosynthetic processes^98^; and **Acadl** (long-chain acyl-CoA dehydrogenase), which is expressed at high levels and catalyzes the initial step of fatty acid β-oxidation. Acadl expression has been shown to be down-regulated by Hif1α in tumor cells^99^. **Mid1ip1** (Mig12, 1.22-fold) by itself or in a complex with **Thrsp** (Spot14, 0.77-fold) interacts with acetyl-CoA carboxylase^100^; increased Mid1ip1 and decreased Thrsp expression leads to higher acetyl-CoA carboxylase activity, which reduces β-oxidation of fatty acids^100^. **Scd1** and **Scd2** (stearoyl-CoA desaturase 1 and 2) were both up-regulated, which would be expected to reduce fatty acid β-oxidation and improve glucose oxidation^101, 102^. Although an increase in stearoyl-CoA desaturase activity might be expected to increase lipid accumulation^102^, reduced expression of **Plin3** (perilipin 3) and **Plin4**, which coat cytosolic lipid droplets^103^, may reflect a reduction in storage of cytosolic lipids in cardiomyocytes due to reduced fatty acid uptake. **Abcd2** (1.61-fold increase) transports very long chain acyl-CoA into peroxisomes and contributes to fatty acid degradation and to synthesis of docosahexaenoic acid (DHA)^104^. Hypoxia has been shown to increase the DHA content of lipid membranes in heart^105^ and, along with eicosapentaenoic acid (EPA), DHA is protective in hypoxia-reoxygenation injury in cardiomyocytes^106^.

### Expression changes indicate reduced use of ATP and substrates for biosynthesis

A number of changes would be expected to reduce the use of ATP and substrates for biosynthesis (Fig. 3D). Reduced expression of **Pcx** (pyruvate carboxylase), which exhibits reduced activity in Drosophila flies adapted to hypoxia^107^, would reduce the conversion of pyruvate to oxaloacetate^108^. The reduction in **Ucp2** (uncoupling protein 2), a member of the hypoxia response GO category that is down-regulated by hypoxia via repression of Pparg^109^, would reduce the transport of oxaloacetate and other 4-carbon intermediates out of mitochondria^110^. Thus, both changes would increase the use of pyruvate and other intermediates for oxidative phosphorylation and reduce their use in ATP-utilizing biosynthetic processes. Reduced expression of **Mlxipl** (ChREBP), which regulates glycolysis and fatty acid synthesis, would also be expected to reduce the use of glucose metabolites for biosynthesis^111^. Two genes involved in glycolysis, **Pgam1** (phosphoglycerate mutase 1) and **Pfkfb2** (6-phosphofructo-2-kinase/fructose-2,6-bisphosphatase 2; 0.77-fold, not shown), were down-regulated. The substrates and products (2-phosphoglycerate, 3-phosphoglycerate, and fructose 2,6-bisphosphate) of the enzymes encoded by these genes serve regulatory functions in glycolysis^112, 113^. Pgam1 is up-regulated in cancer cells and stimulates the use of glycolytic intermediates for biosynthesis^113^, so down-regulation could have the opposite effect. Increased expression of **Eef2k** (eukaryotic elongation factor-2 kinase, 1.31-fold), which reduces consumption of energy by inhibiting protein synthesis during O_2_ deficiency^114^, would also conserve ATP for contraction. **Hspb2**, encoding a heat shock protein that dramatically enhances the efficiency of coupling between ATP hydrolysis and contractile work^115^, was up-regulated (Fig. 3D), suggesting an increase in cardiac efficiency.

### Expression changes indicate altered membrane excitability and contractile function

**Figure 4:**
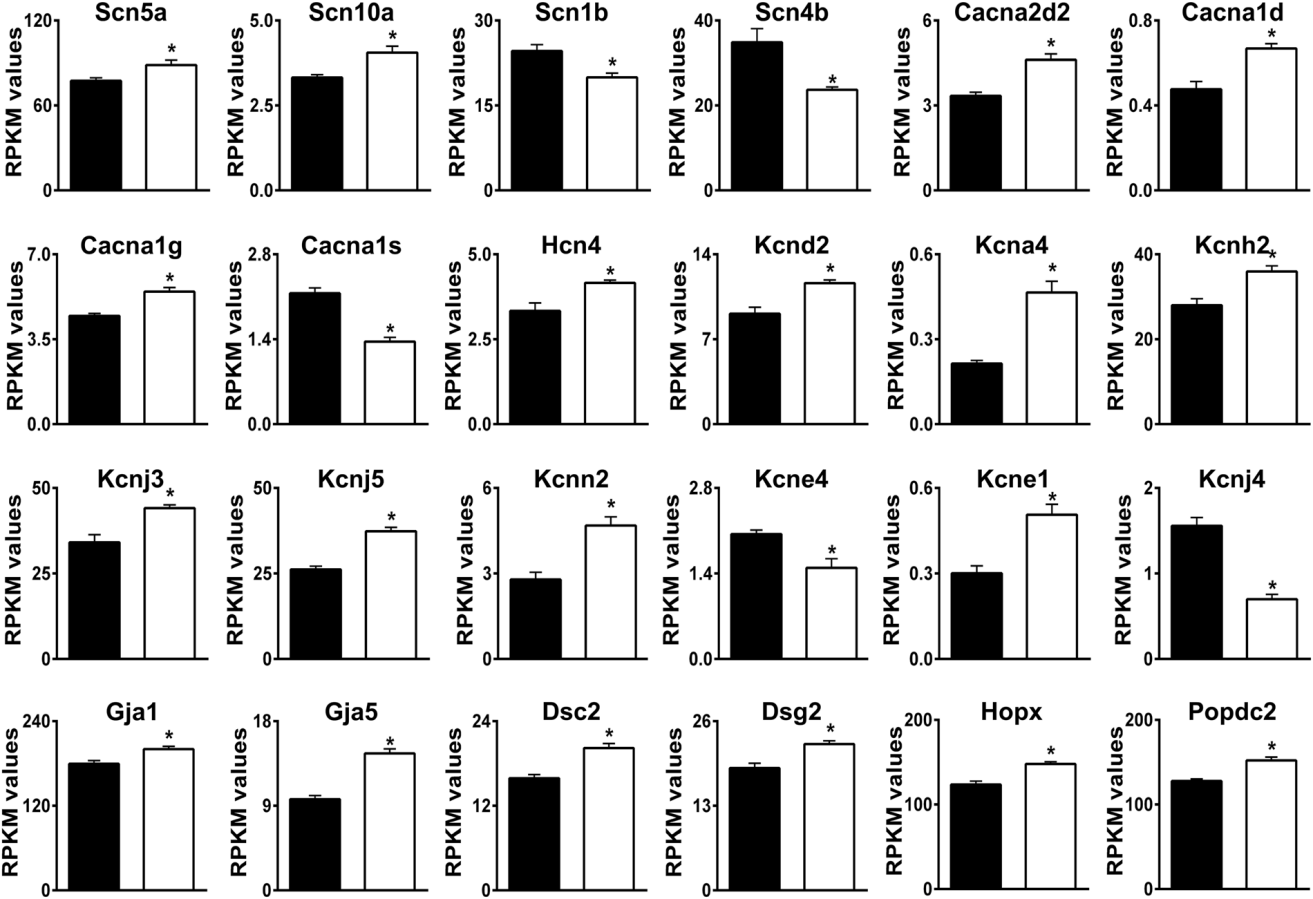
Differential expression of genes involved in membrane excitability and cardiac conduction. Genes relevant to these categories were identified by Gene Ontology analyses. RPKM values for WT (black bars) and AE3-null (white bars) hearts are shown. See Supplementary Information for detailed explanations and references for individual genes. The genes shown are a subset of 104 genes presented in Supplementary Table S5 and 84 transporter, pump, and channel genes in Supplementary Table S6; values are means ± SE; n = 4 for each genotype; *p ≤ 0.01 vs WT controls, except Kcne1 (p = 0.015).

**Figure 5:**
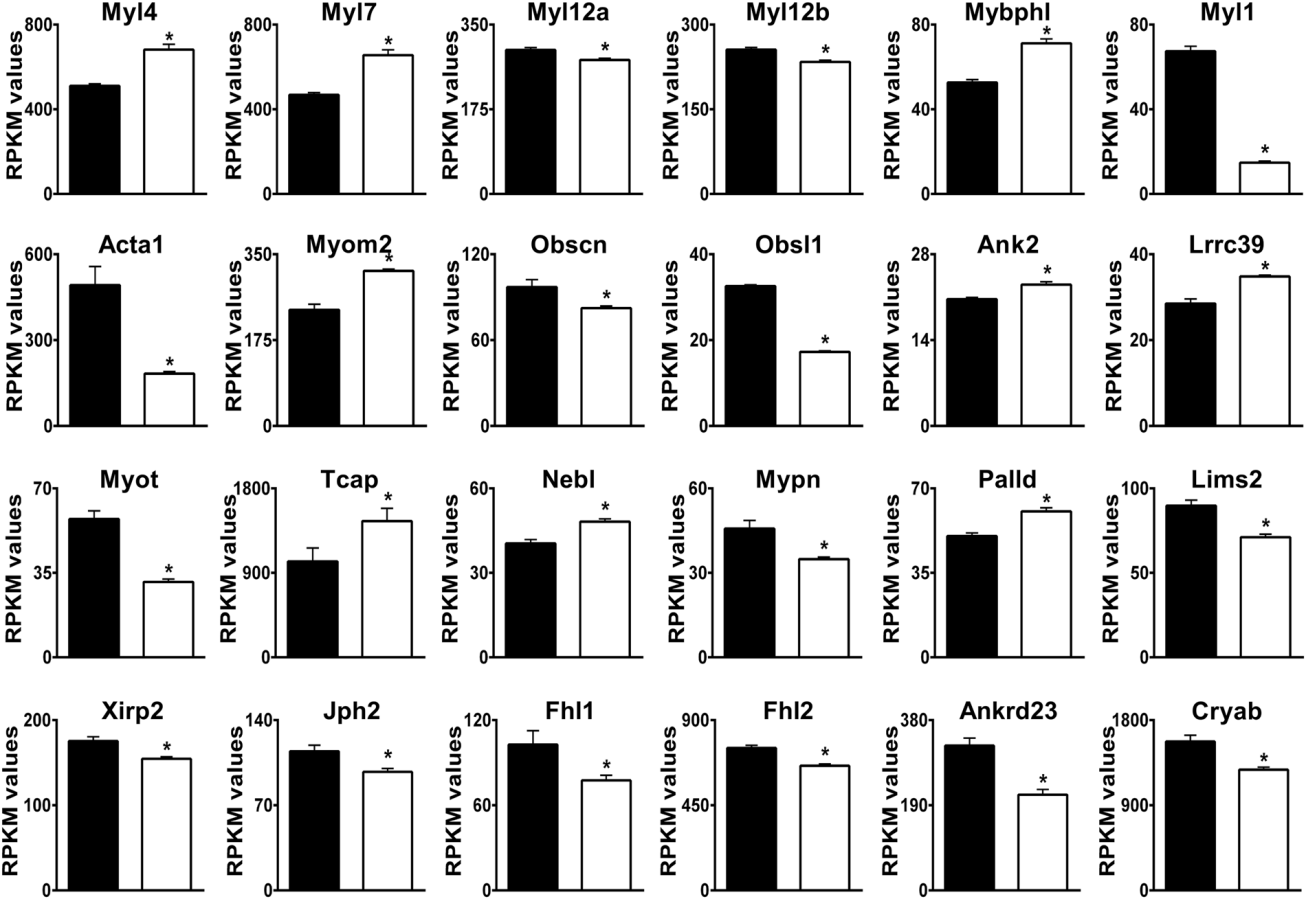
Differential expression of genes encoding sarcomere and sarcomeric cytoskeletal proteins. Genes relevant to these categories were identified by Gene Ontology analyses. RPKM values for WT (black bars) and AE3-null (white bars) hearts are shown. See Supplementary Information for detailed explanations and references for individual genes. The genes shown are a subset of 116 genes presented in Supplementary Table S7. Values are means ± SE; n = 4 for each genotype; *p ≤ 0.01 vs WT controls.

Some particularly prominent groups of differentially expressed genes encoded: (i) proteins that regulate heart rate, membrane excitability, and cardiac conduction (Table 1, Fig. 4), and (ii) myofibrillar proteins localized to the sarcomere, M-band, Z-discs, t-tubules, and intercalated discs (Table 1, Fig. 5). These changes indicate major remodeling of electrophysiological and contractile functions in AE3-null hearts. Although some of these changes may have the potential to provide compensation for deficits resulting from impaired CO_2_ disposal, they do not provide strong evidence for or against any of the three major hypotheses being considered. See Supplementary Results and Discussion for explanations of the functions of specific genes in Figs 4 and 5, and evidence that they represent adaptive rather than pathological changes.

### Expression changes relevant to the Na^+^-loading and pH_i_ regulation hypotheses

**Figure 6:**
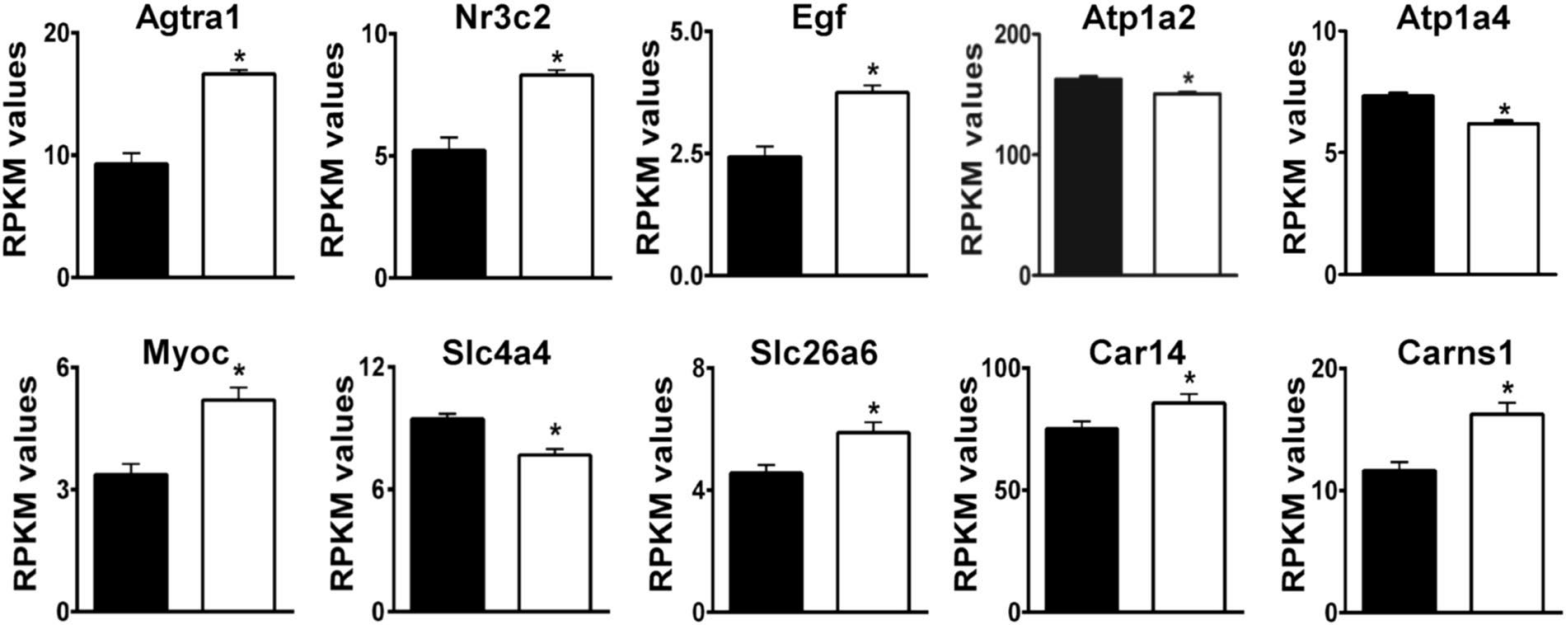
Differential expression of genes with potential for adaptation via Na^+^-loading or regulation of pH_i_. Genes in the top row have the potential to contribute to increased contractility via regulation of Na^+^-loading. Genes in the second row are affected by or involved in intracellular acid-base homeostasis. RPKM values for WT (black bars) and AE3-null (white bars) hearts are shown. Values are means ± SE; n = 4 for each genotype; *p ≤ 0.01 vs WT controls, except Car14 (p = 0.019), which encodes CA XIV, an AE3-interacting protein.

Genes for several proteins that affect contractility by increasing Na^+^-loading and Ca^2+^-loading were sharply up-regulated (Fig. 6). These include **Agtr1a** (angiotensin receptor 1a), **Nr3c2** (mineralocorticoid receptor), and **Egf** (epidermal growth factor), which are involved in a pathway that affects Na^+^- and Ca^2+^-loading^116^. Additional changes consistent with the Na^+^-loading hypothesis were reduced expression of **Atp1a2** and **Atp1a4** (α2 and α4 isoforms of the Na^+^, K^+^-ATPase).

A number of changes appear related to impaired HCO_3_^−^ and pH_i_ homeostasis. **Myoc** (myocilin, 1.52-fold) associates with syntrophins in the dystrophin complex^117^, a component of costameres. Increased myocilin, a member of GO:0014066 (Regulation of Phosphatidylinositol 3-Kinase Signaling), stimulates Akt signaling^117^. Processing and secretion of myocilin is altered by extracellular pH and HCO_3_^−^ concentrations^118^ and could therefore be affected by loss of AE3, which we have shown to cause an increase in Akt signaling in response to elevated heart rates^4^. Thus, changes in myocilin could be a response to localized changes in pH_i_ or HCO_3_^−^ in AE3-null myocytes and may contribute to up-regulation of Akt signaling during pacing.

**Slc4a4** (NBCe1 Na^+^/HCO_3_^−^ cotransporter), the major HCO_3_^−^ uptake mechanism in cardiomyocytes^1, 119^ was down-regulated, which should also reduce Na^+^-uptake, and **Slc26a6**, which mediates both Cl^−^/HCO_3_^−^ exchange and Cl^−^/formate exchange^120^, was up-regulated (Fig. 6). These changes could, in principle, provide compensation for alkalinization resulting from HCO_3_^−^ overload; however, NHE1 protein^4^ and **Carns1** (carnosine synthase) mRNA (Fig. 6) were increased. NHE1 is a powerful H^+^-extrusion mechanism and Carns1 is involved in the synthesis of histidyl dipeptides^121^, which are present at very high levels in cardiac muscle and serve as a major intracellular buffer for H^+^.

**Car14**, which encodes CA XIV, the most abundant carbonic anhydrase in mouse heart, was up-regulated (Fig. 6, see Supplementary Table S8 for expression levels of all CA isoforms). CA XIV is associated with the sarcoplasmic reticulum and colocalizes with mitochondria, where it facilitates CO_2_ venting^13^. CA XIV also binds to AE3 on the extracellular surface of mouse cardiomyocytes, where it catalyzes the conversion of HCO_3_^−^ extruded by AE3 to CO_2_^14^, which would require H^+^ extruded by some other mechanism. Although mitochondrial CA is expressed at very low levels in heart^13^, Car5b, a mitochondrial CA isoform, was significantly increased.

### Potential Cl^−^ and H^+^ extrusion mechanisms to function in AE3-mediated CO_2_ disposal

It has been noted that AE3-mediated HCO_3_^−^ extrusion has the potential to contribute to CO_2_ disposal^10–12^; however, Cl^−^ recycling and parallel H^+^-extrusion would also be needed. In heart, there are many Cl^−^ channels that could mediate Cl^−^ recycling (Supplementary Table S9). The negative membrane potential during most of the excitation-relaxation cycle would allow efficient Cl^−^ recycling, although this would not provide charge balance, and extrusion of a net negative charge would require additional ion transport processes to maintain the resting membrane potential. H^+^-extrusion is particularly problematic because if Na^+^/H^+^ exchange were responsible, the process would not be electrically balanced and extrusion of Na^+^ would require additional expenditure of energy, with an increase in both ATP production and CO_2_ disposal.

The simplest and most energetically efficient H^+^-extrusion mechanism would be the HVCN1 voltage-sensitive proton channel^122^. Although HVCN1 has not been reported previously in cardiac myocytes, data in the EMBL-EBI Expression Atlas (see Methods) indicates that it is expressed in all mammalian tissues. This includes mouse heart and the heart of other mammalian species, including human (Supplementary Table S10). The available expression data indicate that HVCN1 mRNA is expressed in heart at levels comparable to those of NHE1 and also show that AE3 is the most abundant Cl^−^/HCO_3_^−^ exchanger in mammalian hearts.

## Discussion

Although AE3 was identified almost 30 years ago and shown to be expressed at high levels in heart^123, 124^, its physiological functions are not yet established. Here we used RNA Seq analysis to assess the major hypotheses about its functions in cardiac muscle. The differential expression data provide strong support for the CO_2_ disposal hypothesis and suggest major avenues of investigation that could be used to further test this hypothesis. This is potentially important as CO_2_ disposal is generally thought to occur entirely by diffusion, either directly across the plasma membrane or through gas channels^125, 126^. However, transporters involved in transepithelial ion transport processes are able to extrude large quantities of HCO_3_^−^ and H^+^ that are derived from CO_2_ (see Supplementary Results and Discussion), so transport-mediated CO_2_ disposal is a reasonable mechanism. Also, the prior demonstrations of carbonic anhydrase-mediated CO_2_ hydration in the venting of CO_2_ from cardiomyocyte mitochondria^13^, which is needed to avoid inhibition of oxidative phosphorylation, and the association of CA XIV with the extracellular domains of AE3^12, 14^ are consistent with transport -mediated CO_2_ disposal in heart.

The differential expression patterns most strongly indicating impaired CO_2_ disposal in AE3-null cardiomyocytes were the changes in genes mediating hypoxia responses, coupled with changes that would likely increase blood flow, and reduced expression of angiogenesis genes, all of which indicated impaired O_2_/CO_2_ balance in heart. The changes in angiogenesis genes that play direct roles in vascular tissues, while modest, were unambiguously consistent with a reduction in angiogenesis in AE3-null hearts, which initially seemed inconsistent with the apparent hypoxia response. However, the hypoxia response is not due to systemic hypoxia, as global loss of AE3 does not affect respiratory function, systemic acid-base homeostasis, or blood gasses (O_2_, CO_2_)^127^. Also, the level of blood lactate, which is utilized as an energy source by cardiac myocytes, is slightly reduced^127^ rather than increased as in systemic hypoxia. Furthermore, systemic hypoxia should increase, not decrease, angiogenesis. The data are consistent with a mild hypoxia response in AE3-null myocytes, which in turn secrete vasodilators in order to increase blood flow. Increased oxygenation of the stromal tissue would lessen the hypoxia in myocytes but would also reduce the stimulus for angiogenesis. These results are consistent with impaired O_2_/CO_2_ balance occurring specifically in cardiomyocytes, as would be expected if AE3-mediated extrusion of HCO_3_^−^ plays a major role in CO_2_ disposal.

The RNA Seq data provide limited support for the Na^+^-loading hypothesis. Activation of Agtr1a^128^ and treatment with Egf^129^ stimulate contractility in cardiac myocytes and isolated hearts. Increased contractility in response to myocardial stretch requires angiotensin, mineralocorticoid, and Egf receptor activities^130^, along with the downstream effector NHE1, which was up-regulated at the protein level in AE3-null hearts^4^. Activation of this pathway is known to increase Na^+^- and Ca^2+^-loading^116^ and, as proposed previously^2, 6–8^, when NHE1 activity is activated to increase Na^+^-loading, H^+^-extrusion could be balanced by AE3-mediated HCO_3_^−^ extrusion. This function is compatible with a CO_2_ disposal function, since AE3 could be involved in maintaining pH_i_ balance in response to H^+^-extrusion by either NHE1 or HVCN1. Nevertheless, in previous studies^3, 4^ we observed no changes in Ca^2+^-handling that might explain the reduction in force-frequency response in AE3-null mice, and the expression changes observed here provide only limited support for the Na^+^-loading hypothesis.

The current data and previous studies do not support the hypothesis that the major function of AE3 is to mediate recovery from an alkaline load *in vivo*, even though AE3-mediated recovery from an alkaline load can be demonstrated following experimental manipulations *in vitro* ^2^. NHE1 protein was increased in AE3-null hearts^4^ and NHE1 mRNA was increased in isolated AE3-null cardiac myocytes^2^. Also, mRNA encoding Carns1, involved in histidyl dipeptide synthesis, was increased. Histidyl dipeptides would likely be important for efficient venting of CO_2_ from mitochondria^13^ as they would buffer H^+^ produced by CO_2_ hydration, thus facilitating the reaction that converts waste CO_2_ to H^+^ and HCO_3_^−^. Increased expression of NHE1 and Carns1 mRNA suggest that the loss of AE3 leads to an increased need for NHE1-mediated H^+^-extrusion and H^+^ buffering capacity. Given that AE3 extrudes HCO_3_^−^, this may seem paradoxical; however, CO_2_ hydration generates equimolar amounts of HCO_3_^−^ and H^+^ and a reduction in HCO_3_^−^ extrusion could affect not only the rate of CO_2_ hydration, but also a parallel mechanism of H^+^ extrusion that is affected by extracellular carbonic anhydrase associated with AE3 (discussed below).

The up-regulation of Car14 is potentially relevant, as CA XIV has been proposed to play an important role in both CO_2_ venting from mitochondria^13^ and in AE3-mediated HCO_3_^−^ extrusion from cardiac myocytes, where it is associated with the extracellular domains of AE3^14^. CA XIV also associates with AE3 in retina and brain^12, 14^. Also, its mRNA was increased in neurons of AE3-null mice^14, 131^, consistent with a deficit in CO_2_ disposal in AE3-null neurons. This could be responsible for the epilepsy phenotype in AE3-null mice^14, 132^ and in humans with a heterozygous AE3 mutation^133^, as hypoxia can contribute to epilepsy^134^. Interestingly, expression of Car5b, a mitochondrial CA isoform, was increased in AE3-null hearts, suggesting a perturbation of CO_2_ venting from mitochondria. This is consistent with the reduction in AE3-mediated extrusion of HCO_3_^−^ from the cell, which would shift the equilibrium toward a reduction in CO_2_ hydration. These changes support the CO_2_ disposal hypothesis.

Because a reduction in carbonic anhydrase-mediated hydration of waste CO_2_ as it exits the mitochondria causes an inhibition of oxidative phosphorylation^13^, it is reasonable to expect that a reduction in the ability to dispose of HCO_3_^−^ would elicit adaptative changes in energy metabolism. The changes in metabolic genes, while modest, suggest an increase in glucose metabolism and a reduction in fatty acid uptake and metabolism, which would provide a more favorable ATP/O_2_ ratio during mild hypoxia. Interestingly, a number of the proteins involved in glucose metabolism are affected by activation of Akt (indicated in Fig. 3), which is known to have a major effect on glucose metabolism in heart^135^. The increased Akt phosphorylation observed when AE3-null mice were subjected to atrial pacing^4^ and upregulation of glucose metabolism genes that respond to Akt suggest that glucose metabolilsm may be stimulated in AE3-null hearts during acute biomechanical stress, when O_2_ utilization would be increased. In addition to an improved ATP/O_2_ ratio, a shift in the relative balance between glucose and fatty acid metabolism would also be expected to improve cardiac function due to a reduction in the negative effects of fatty acid metabolism on cardiac efficiency^136^. An increase in cardiac efficiency is supported by the up-regulation of Hspb2 (Fig. 3D). When challenged with β-adrenergic stimulation, mice lacking Hspb2 hydrolyzed more ATP but performed less work^115^. Thus, an increase in Hspb2 should provide better protection of energy reserves and improved contractility in response to β-adrenergic stimulation and other stress conditions. In addition, there were a number of expression changes that would be expected to reduce the use of substrates and ATP for biosynthesis, which would conserve energy for muscle contraction. A reduction in use of ATP and substrates for biosynthesis is consistent with the smaller hearts in AE3-null mice^2, 4^.

**Figure 7:**
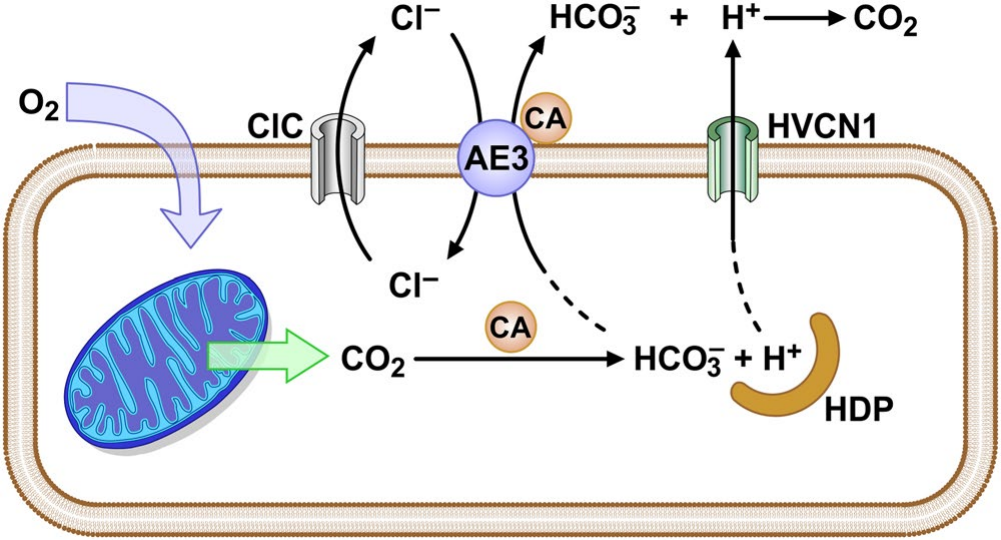
Model for the role of the AE3 Cl^−^/HCO_3_^−^ exchanger in transport-mediated CO_2_ disposal. Oxygen entering the myocyte is rapidly converted to CO_2_ in mitochondria. CO_2_ venting from mitochondria^13^ is facilitated by CA-mediated conversion of CO_2_ to HCO_3_^−^ and H^+^, with H^+^ buffered by histidyl dipeptides (HDP) and other components, thereby effectively blocking the back reaction by keeping the concentration of free H^+^ low. CO_2_ disposal is proposed to be mediated by a combination of HCO_3_^−^ extrusion by AE3, Cl^−^ recycling via Cl^−^ channel activity, H^+^-extrusion via HVCN1 during each depolarization, and extracellular carbonic anhydrase (CA) activity to generate CO_2_.

The major difficulty in proposing a role for AE3 in transport-mediated CO_2_ disposal in cardiac myocytes is that it would require the parallel operation of an energetically-efficient H^+^ disposal mechanism. Of the known mechanisms of H^+^ extrusion, only the HVCN1 H^+^ channel, which is expressed in heart (Supplementary Table S8) and seems to be ubiquitous in mammalian tissues, would appear to have the necessary properties. It is perfectly selective for H^+^, mediates outward transport only, and is strongly activated by intracellular acidity and a positive membrane potential^122^. As illustrated in Fig. 7, HVCN1 and AE3 have the potential to form an efficient mechanism for transport-mediated CO_2_ disposal. Because AE3 is electroneutral and unaffected by changes in membrane potential, its HCO_3_^−^ extrusion activity is driven by the inwardly directed Cl^−^ gradient. With recycling of Cl^−^ through sarcolemmal Cl^−^ channels while the cell is in the polarized resting phase, efficient export of HCO_3_^−^ being produced via CO_2_ hydration^13^ would be maintained. H^+^ passing through HVCN1 during each action potential would be catalytically combined with HCO_3_^−^ present in the unstirred layer via extracellular CA XIV that is associated with AE3^12, 14^, thereby preventing a buildup of acid on the cell surface. It should be noted that generation of CO_2_ by this mechanism would not require tight coupling in which the HCO_3_^−^ being extruded by AE3 is directly combined with H^+^ being extruded by HVCN1. With HCO_3_^−^ serving as the major extracellular buffer and AE3 continuously replenishing HCO_3_^−^ being consumed by CO_2_ production via extracellular CA activity, the HCO_3_^−^ concentration in the unstirred layer would be maintained and the CO_2_ generated would be washed away in the blood.

Although additional studies will be needed to test various aspects of the proposed CO_2_ disposal mechanism and to assess the metabolic, electrical, and contractile consequences of its perturbation, the expression data described here suggest that AE3 plays a central role in transport-mediated CO_2_ disposal. Transport-mediated CO_2_ disposal occurs in epithelial tissues as a side effect of acid-base and electrolyte transport; however, this is the first description of an energetically-efficient system for transport-mediated CO_2_ disposal. In addition, the data set (included in its entirety in Supplementary Files) provides both a framework and a rich source of additional information for further investigations of the cardiac functions of AE3.

## Methods

### RNA Seq Analysis

Total RNA was isolated from whole hearts of 4-month-old FVB/N WT and AE3-null male mice (n = 4 of each genotype). All procedures using animals conformed to guidelines published by the National Institutes of Health (Guide for the Care and Use of Laboratory Animals) and were approved by the Institutional Animal Care and Use Committee at the University of Cincinnati. Samples were subjected to RNA Seq analysis in the University of Cincinnati Genomics and Sequencing Core using the Illumina HiSeq. 1000 platform. Sequence reads were aligned to the reference mouse genome using TopHat aligner^137^. The full data set was deposited in the Gene Expression Omnibus (GEO accession number GSE70471) and is also provided as an Excel File in Supplementary Table S1. Additional Excel Files with subsets of expression data are provided in Supplementary Tables S2–S7. Fold-change expression data in the Excel files relate expression in AE3-null hearts relative to that observed in WT hearts.

Statistical analysis to identify differentially expressed genes was performed using the negative-binomial model of read counts as implemented in DESeq Biocondoctor package^138^. Most of the genes (80%) included in the figures had an FDR (False Discovery Rate) < 0.05, which is a measure of the probability of a false positive given the inclusion of over 23,000 genes in the analysis. Among the 536 genes with FDR < 0.05, 284 were up-regulated and 252 were down-regulated. Some genes with FDR > 0.05 were also included because they were in significantly enriched GO categories that were a major part of the phenotype (see Table 1 and legends for Supplementary Tables S2–S7). Inclusion of genes with a significant P value but an FDR > 0.05 that were also present in significant GO categories, which were derived from a small subset of genes, protected against exclusion of false negatives. mRNA expression is presented as Reads Per Kilobase per Million mapped reads (RPKM), which normalizes for the size of the mRNA and provides a measure of the relative abundance of specific transcripts. Approximately 25 million reads were achieved per sample.

Cardiac RNA Seq expression data for mammalian hearts (Supplementary Table S10) and other tissues was obtained from the European Bioinformatics Institute (EBI) Expression Atlas (http://www.ebi.ac.uk/gxa/home). Heart data was for mRNA expression in hearts of both male and female Fisher 344 rats at 4 developmental stages^139^ and in hearts of C57Bl6 mice, opossum, rhesus monkey, and human^140^. The latter study used a normalization procedure that allowed cross-species comparisons of specific genes.

### Gene Ontology Analysis

The online GOrilla program^15^ (Gene Ontology enRIchment anaLysis and visuaLizAtion tool; http://cbl-gorilla.cs.technion.ac.il/) was used for Gene Ontology analyses. This program can be accessed and used without registering and is self-explanatory and easy to use. Two analysis options were used: (**1**) Single Rank List in which the entire gene set was ranked according to P values and (**2**) Two Unranked Lists, in which a target list of genes with a P value or FDR (false discovery rate) value within a specified range was compared against the background list of all genes (over 23,000 total, with 18,892 expressed genes) to which reads were mapped. Neither analysis option alone was completely satisfactory for identifying relevant GO categories and genes. Thus, we used both the Single Rank option, which considers only the relative significance ranking of all genes and does not depend on an arbitrary cutoff and the Two Unranked lists option, in which various cutoffs, ranging from very stringent (FDR < 0.01–0.05) to less stringent (P < 0.01) were used. The Single Rank option identified some functions that were highly enriched at the top end of the significance range and other functions that were significantly enriched over a very large range, but with lower enrichment scores. Both approaches are needed to minimize inclusion of false positives or exclusion of false negatives.

Enrichment analysis for both options uses the hypergeometric distribution to calculate statistical probabilities^15^. The Single Rank option avoids setting an arbitrary cutoff of P values to be considered and was particularly useful for identifying GO categories that were highly enriched at the top end of the significance range for individual genes. When using the Single Rank analysis, all genes were ranked by P values and the distribution of genes in each GO category was used to calculate significance scores and enrichment of specific GO categories. When using the Two List analysis, significance and enrichment in each GO category is calculated based on the number of genes for specific GO categories appearing in the target list and background list. Enrichment (N, B, n, b) is defined as follows: N is the total number of genes, B is the total number of genes associated with a specific GO term, n is the number of genes in the top of the input list or in the target set when appropriate, b is the number of genes in the intersection; Enrichment = (b/n)/(B/N). As shown in Supplementary Fig. S2, the program provides a visual display of hierarchically arranged GO categories, with large broad categories displayed at the top and smaller, more specific categories nested within one or more of the broad categories. In addition, the program provides a list of GO categories ranked by significance and a list of the genes in each GO category, which can be shown or hidden. When performing GOrilla analysis, the genes were not separated into up-regulated or down-regulated genes since a given process might be affected positively (or negatively) by up-regulation of some genes and down-regulation of others. Significant GO categories were inspected, grouped by related functions, and a list of non-redundant genes for each broad category was prepared as described in the legends for Supplementary Tables S2–S7. In some cases, particularly (1) Hypoxia, Vasodilation, Angiogenesis (Supplementary Table S2) and (2) Energy Metabolism (Supplementary Table S4), additional genes were included in the lists based on PubMed literature searches, which relied heavily on PubMatrix. Those searches can be publicly accessed as described below.

### PubMatrix Analyses

Pubmatrix (https://pubmatrix.irp.nia.nih.gov/), an online literature search tool^16^, was used to search PubMed to identify relevant publications describing the functions of specific genes in various biological processes (Supplementary Fig. S3). In each run, PubMatrix performs pairwise literature searches of up to 100 search terms and up to 10 modifier terms. The search terms corresponded to the 536 genes with an FDR ≤ 0.05; for each gene, the gene symbol was grouped with common names for the gene. For example, search terms for the angiotensin II receptor, type 1 was: (Agtr1a or AT1a or Agtr1). The 536 genes with FDR < 0.05 were grouped into 4 sets of up-regulated genes (Sets 1–4) and 4 sets of down-regulated genes (Sets 5–8) and each set was searched against modifier terms for 1) Hypoxia and related processes [Modifier terms: (hypoxia or hypoxic), (HIF1 or Hif1alpha or Hif1a or Hif), (Egln3 or PHD3), (Epas or Hif2a), (Vegf or Vegfa), Angiogenesis, Vasodilation, Vasoconstriction, (ischemia or ischemic), (heart or cardiac)] and 2) Energy Metabolism [Modifier terms: “energy metabolism”, “lipid metabolism”, “beta oxidation”, (mitochondria or mitochondrial), “glucose metabolism”, “glucose oxidation”, Glucose, Glycolysis, Ampk, AKT]. As illustrated in Supplementary Fig. S3, each set of searches is displayed on a grid that allows any pairwise search to be opened; the searches are stored and can be reopened at any time. The reader can access the searches performed here for Hypoxia and Metabolism genes (under Public Results, username Vair&Shull) by registering on PubMatrix with a username (typically email address) and a simple password.

### Quantitative Real-time PCR (RT-PCR) analysis

Total RNA was isolated from hearts of 4-month-old FVB/N male mice (N = 4 of each genotype) using Tri-reagent (Molecular Research Center, Cincinnati, OH). cDNA was prepared by random priming using Superscript III First-strand synthesis kit from Life Technologies.

RNA Seq data were validated by quantitative PCR (Supplementary Fig. S4). RT-PCR analysis to determine mRNA levels of differentially expressed genes selected from each of the categories discussed was performed on an ABI 7300 Real Time PCR system according to the manufacturer recommended protocol using both SYBR green and TaqMan assays. The primer sequences used for SYBR green-based fluorescence were Gapdh: 5′-AGGTCGGTGTGAACGGATTTG-3′ and 5′-TGTAGACCATGTAGTTGAGGTCA-3′; Hif1a: 5′-ACCTTCATCGGAAACTCCAAAG-3′ and 5′-CTGTTAGGCTGGGAAAAGTTAGG-3′; Cirbp: 5′-GGACTCAGCTTCGACACCAAC-3′ and 5′-ATGGCGTCCTTAGCGTCATC-3′; Rbm3: 5′-CTTCGTAGGAGGGCTCAACTT-3′ and 5′-CTCCCGGTCCTTGACAACAAC-3′; Egln3: 5′ GGCTGGGCAAATACTATGTCAA 3′ and 5′-GGTTGTCCACATGGCGAACA-3′; Tbx5: 5′-TTGGATGAGGTGGAGAGAGC-3′ and 5′-ACACAGGATGTCTCGGATGC-3′; Adm: 5′-AACCAGCTTCATTCTGTGGC-3′ and 5′-TGGACTTTGGGGTTTTGCTA-3′; Slc26a6: 5′-GTGGCGAACTTGGTTCCGAT-3′ and 5′-AGCCATTCACGCACAGGATAC-3′.

TaqMan assays were purchased from Life Technologies. The assay IDs for the TaqMan assays for Gapdh, Adra1b, Kcnj5, Gja5, Myl7, Myl1, Myot, Agtr1a, Carns1, are Mm99999915_g1, Mm00431685_m1, Mm01175829_m1, Mm00433619_s1, Mm01183005_g1, Mm00659043_m1, Mm00498877_m1, Mm01957722_s1, and Mm01236521_m1 respectively. The ΔΔCt method was used to calculate relative expression of analyzed transcripts after normalization with Gapdh in both cases. Values are means ± SE and significance was determined by Student’s t-test.

## Electronic supplementary material

Supplementary Information

Dataset 1

Dataset 2

Dataset 3

Dataset 4

Dataset 5

Dataset 6

Dataset 7
