# Supplementary material for: RNA SEQ Analysis Indicates that the AE3 Cl−/HCO3− Exchanger Contributes to Active Transport-Mediated CO2 Disposal in Heart
Source: Sci Rep. 2017 Aug 4;7:7264. doi: 10.1038/s41598-017-07585-y (PMC5544674; doi:10.1038/s41598-017-07585-y)
Supplement: Supplementary file 1 — Supplementary Information [file 41598_2017_7585_MOESM1_ESM.pdf]

Supplementary Information

RNA SEQ Analysis Indicates that the AE3 Cl^-/HCO_3^- Exchanger Contributes to Active Transport-Mediated CO_2 Disposal in Heart

Kanimozhi Vairamani[1], Hong-Sheng Wang[2], Mario Medvedovic[3], John N. Lorenz[4], and Gary E. Shull[1]

Departments of [1]Molecular Genetics, Biochemistry and Microbiology; [2]Pharmacology and Cell Biophysics; [3]Environmental Health; and, [4]Cellular and Molecular Physiology, University of Cincinnati College of Medicine, Cincinnati, Ohio, 45267 USA

Supplementary Results and Discussion…..2

Comments on RNA Seq Analysis……………………………………………………..2

Comments on Gene Ontology and PubMatrix Analyses……………………………...2

Hypotheses proposed for the physiological functions of AE3………………………...3

Changes involved in membrane excitability and cardiac conduction…………………5

Changes involving the sarcomere and sarcomeric cytoskeleton………………………6

Additional changes that might reduce passive tension/stiffness………………………8

Evidence that changes are largely adaptive rather than pathological…………………8

References…………………………………………………………………………….……10

Supplementary Figures S1-S4..........…………………………………………………….15

Legends for Supplementary Tables S1-S7 online (Excel Files of RNA Seq data)………………19

Supplementary Tables S8-S10...…………………………………………………………23

Supplementary Results and Discussion

Comments on RNA Seq analysis. RNA Seq analysis was used to identify differential mRNA expression patterns in hearts of highly inbred FVBN WT and AE3-null mice. The expression changes were modest, but variability between samples of each genotype was remarkably low and over 500 genes had a False Discovery Rate (FDR) < 0.05. The entire data set, which queried over 23,000 genes, with just under 19,000 genes showing expression in whole heart, is presented as an Excel file in Supplementary Table S1 online. Additional Excel files present subsets of genes as described in the legends for Supplementary Tables below. These include: Supplementary Table S2 online, Hypoxia, Vasodilation, and Angiogenesis; Supplementary Table S3 online, Signaling proteins; Supplementary Table S4 online, Energy Metabolism; Supplementary Table S5 online, Membrane Excitability and Cardiac Conduction; Supplementary Table S6 online, Transporters, Pumps, and Channels; Supplementary Table S7 online, Sarcomere and Sarcomeric Cytoskeleton proteins. These Excel files provide interested readers with a comprehensive listing of the gene expression changes occurring in AE3-null hearts that may allow further analyses of the hypotheses described below or provide additional insights into the biological functions of AE3.

When appropriately sorted, these files also provide a listing of relative expression levels (normalized to the size of the mRNA; RPKM values) for genes expressed in the FVBN mouse heart, which can be used to assess relative mRNA expression of various proteins or isoforms.

Comments on Gene Ontology and PubMatrix analyses to identify relevant genes. The Gene Ontology classification used by the GOrilla program is that of the Gene Ontology Consortium, which is supported by the National Human Genome Research Institute. The assignment of genes to various Gene Ontology (GO) categories is being constantly updated and provides only a rough guideline about processes and functions that are affected by the experimental perturbation. Most genes are assigned to multiple GO categories and the assignments are often made on the basis of incomplete information. Because AE3 is active primarily in cardiac myocytes, identification of processes and functions that are affected by loss of AE3 in heart is made even more difficult by the presence of many different cell types in heart tissue, some of which may exhibit secondary responses to changes originating in myocytes. Of particular relevance to the CO_2 disposal hypothesis, the Response to Hypoxia GO category also contained genes present in Angiogenesis GO categories, and the direction of changes were often inconsistent or contradictory. However, PubMatrix literature analyses revealed that angiogenesis genes with functions that are largely restricted to vascular tissue were consistently changed in a direction opposite to what would be expected in tissue hypoxia, thus indicating well-oxygenated stromal tissue. Despite changes suggesting a reduction in angiogenesis, expression of many other genes was changed in a direction consistent with hypoxia. As discussed in the text, however, these findings are consistent with the CO_2 disposal hypothesis.

The need for hypotheses to guide the evaluation of complex RNA Seq data can be illustrated by the fact that changes in hypoxia and angiogenesis genes were not as obvious as the changes in genes for cardiac electrical and myofibrillar functions, which indicated major remodeling of these systems. Thus, in the absence of specific hypotheses about the functions of AE3, indications of a perturbation in O_2/CO_2 balance would likely not have seemed as relevant as the changes in genes encoding channels and myofibrillar proteins. Changes in channel expression could, for example, lead one to propose that these effects are due to perturbations of intracellular anion concentrations or subsarcolemmal pHi, rather than remodeling, and this remains a formal possibility. For example, as noted in the legend for Supplementary Table S9,

changes in the expression of several Cl^- channels likely represent remodeling in response to reduced Cl^- uptake resulting from the loss of AE3.

Hypotheses that have been proposed for the physiological functions of AE3. On the basis of biological evidence and the tissue distribution and transport activities of AE3, three major hypotheses about its physiological functions, which are not mutually exclusive, have been proposed. Here we discuss the relative merits of these hypotheses based on information available before the RNA Seq experiments were performed.

1. CO_2 disposal hypothesis: Removal of CO_2 from cells is generally thought to occur by diffusion directly across the plasma membrane or through gas channels[1-4]. Although there is vigorous debate and controversy about which route is more important[1-4], diffusion undoubtedly contributes to CO_2 disposal in many tissues; however, it is unlikely to be the only mechanism.

The possibility of transport-mediated CO_2 disposal is strongly supported by several dramatic examples of CO_2 extrusion from epithelial cells in the form of HCO_3^- and H^+. Gastric acid secretion involves apical H^+ extrusion via the H^+,K^+-ATPase and basolateral HCO_3^- extrusion via the AE2 Cl^-/HCO_3^- exchanger[5]. HCO_3^--recovery in the renal proximal tubule (~4,500 mmoles/day in an average human) involves basolateral NBCe1-mediated HCO_3^- extrusion and apical NHE3-mediated H^+ extrusion[6-8]. In both cases, CO_2 is hydrated via intracellular carbonic anhydrase and the hydration products are removed from the cell by active transport mechanisms. Although these processes are not for the function of CO_2 disposal per se (and would not be energetically efficient if this were their primary function), they illustrate the potential of HCO_3^- and H^+ extrusion as an effective means of CO_2 disposal. AE3 is expressed at very high levels in cardiac muscle relative to other Cl^-/HCO_3^- exchangers[9], and would appear to be well-suited to mediate bulk extrusion of HCO_3^- from cardiac myocytes.

A specialized case of CO_2 disposal involving Cl^-/HCO_3^- exchange is the facilitation of HCO_3^-/CO_2 transport in the blood by the Band 3 (AE1) Cl^-/HCO_3^- exchanger[7,8]. After hydration of CO_2 that diffuses into the red blood cell, it is extruded from the cell in the form of HCO_3^-; however, H^+ remain in the cell and are buffered by hemoglobin rather than being extruded via active transport as in the examples given above. This allows CO_2 to be reformed as blood passes through the lungs and O_2 binding to hemoglobin displaces bound H^+.

Transport-mediated CO_2 disposal, referred to as "CO_2 siphoning", was first proposed for glial cells of the salamander retina[10], which exhibit high O_2 consumption. An electrogenic Na^+-HCO_3^- cotransporter (NBC) with a Na^+:HCO_3^- ratio of 1:3, similar to that of NBCe1 in the renal proximal tubule, was identified as a possible HCO_3^- extrusion mechanism. The H^+ extrusion mechanism was not identified, but the author speculated that it might be a Na^+/H^+ exchanger[10]. However, such a CO_2 disposal system would not be energetically efficient. With these specific transporters, if HCO_3^- and H^+ extrusion were balanced, each cycle of NBC/NHE activity would extrude 2 negative charges and take up 2 Na^+, which would require expenditure of ATP via Na^+,K^+-ATPase activity to maintain the membrane potential and ion balance. Kopito, Casey, and colleagues were the first to note that AE3-mediated HCO_3^- extrusion has the potential to contribute to CO_2 disposal in retina and neuronal cells[11-13]. However, a H^+ extrusion mechanism that allows maintenance of overall charge and ion balance, which would be essential for energetically efficient transport-mediated CO_2 disposal, was not identified.

A landmark study dealing with acid-base homeostasis in cardiac myocytes, that is relevant to both the CO_2 disposal hypothesis and the other hypotheses discussed below, is the work of Schroeder et al[14]. These investigators showed that CO_2 generated by oxidative phosphorylation in cardiomyocytes is rapidly hydrated to H^+ + HCO_3^- to facilitate CO_2 venting

from mitochondria. Inhibition of carbonic anhydrase (CA) showed that intracellular hydration of CO_2, a metabolic waste product, was essential to prevent inhibition of oxidative phosphorylation. CA-catalyzed hydration of $CO_2 \rightarrow HCO_3^-$ and H^+ in cardiomyocytes (and in the other metabolically highly active cells mentioned above) is a far-from-equilibrium reaction. During oxidative phosphorylation, waste CO_2 is generated continuously and at high levels and converted to $HCO_3^- + H^+$, with H^+ being buffered by histidyl dipeptides and other components. Although one might question whether CO_2, after having been converted to $HCO_3^- + H^+$ as it exits the mitochondria, might reform in another cytosolic sub-compartment and exit the cell by diffusion, the extremely high volume density of mitochondria in cardiac myocytes, ranging from 25% (human) to 38% (mouse)[15], and prevention of the back reaction by buffering of H^+ by histidyl dipeptides and other components makes this unlikely. Clear implications of this study are that: 1) equimolar amounts of $HCO_3^- + H^+$ are generated from the CO_2 produced by oxidative phosphorylation, and 2) the myocyte must dispose of both hydration products in order to facilitate ongoing hydration of the CO_2 being produced.

A final consideration supporting a role for AE3 in CO_2 disposal is the demonstration by Casey and Alvarez that extracellular CA XIV is physically associated with AE3 in excitable tissues, including retinal cells, neurons, and cardiac myocytes[13,16]. These investigators noted that "Association of AE3 and CA XIV may represent a mechanism to enhance disposal of waste CO_2". Because AE3 extrudes HCO_3^-, extracellular CA activity would be expected to catalyze the conversion of HCO_3^- to CO_2, a reaction that would also require H^+. HVCN1 voltage-sensitive H^+ channel activity is a likely source of H^+ for this reaction.

2. Stimulation of Na^+-loading and Ca^{2+}-loading hypothesis: As reviewed by Garciarena et. al.[23], Na^+-loading via NHE1-mediated Na^+/H^+ exchange and to a much lesser degree by Na^+-HCO_3^- cotransport is well-established and can be sharply increased by up-regulating their activities. The increase in Na^+ stimulates Ca^{2+}-loading via the NCX1 Na^+/Ca^{2+} exchanger, with subsequent effects on contractility[17]. The hydration of CO_2 that occurs to facilitate CO_2 venting from mitochondria[20] suggests that Na^+-loading via NHE1 is largely dependent on H^+ derived from CO_2 hydration. This implies that parallel HCO_3^- extrusion would also be required as it would be necessary to remove both products of CO_2 hydration to prevent inhibition of ongoing CO_2 hydration and to maintain pH_i homeostasis. Parallel Cl^-/HCO_3^- exchange, which serves as an "acid-loading" mechanism that balances acid-extrusion, has been proposed as a means of driving NHE1 activity in a pH-neutral manner. AE3 has been suggested as the most likely isoform to mediate this activity[18-21], although other Cl^-/HCO_3^- exchangers could contribute to this process or provide some compensation in AE3-null mice.

The fact that the degree of Na^+-loading can be stimulated under various conditions[17] implies that either **1**) the production of H^+ can be varied depending on the need for Na^+-loading or **2)** the extrusion of H^+ occurring via an alternate Na^+-independent mechanism can be shifted to the NHE1 Na^+/H^+ exchanger to increase Na^+-loading. The ubiquitous HVCN1 voltage-gated H^+ channel can, in fact, mediate Na^+-independent H^+-extrusion, thus indicating the potential for regulating the relative amounts of H^+ extrusion via each route according to the need for Na^+-loading. Either route would appear to require parallel HCO_3^- extrusion, which is carried out in heart via Cl^-/HCO_3^- exchange.

Na^+-loading and Ca^{2+}-loading is known to cause cardiac hypertrophy, so the reduced heart weight/body weight ratios in AE3-null mice[20,22], the reduction in Ca^{2+}-loading in isolated myocytes from AE3-null mice that also lack the NKCC1 Na^+-K^+-$2Cl^-$ cotransporter[22], and the reduction in angiotensin-induced hypertrophy in isolated AE3-null myocytes[20] are consistent

with the hypothesis that AE3 can facilitate Na^+-loading. Furthermore, it is possible that the impaired force-frequency response in AE3-null mice subjected to atrial pacing[23] could be due, at least in part, to a reduction in Na^+- and Ca^{2+}-loading *in vivo*. Nevertheless, Ca^{2+}-transients in isolated AE3-null myocytes were normal and AE3 ablation had no effect on the degree of hypertrophy in a hypertrophic cardiomyopathy model[24], which argues against a critical role for AE3 in Na^+- and Ca^{2+}- loading for which adequate compensation cannot occur.

3. Recovery from an alkaline load hypothesis: Experiments in which mouse AE3-null and wild-type myocytes were subjected to an alkaline load show that AE3 mediates 60-70% of the maximum Cl^-/HCO_3^- exchange activity in isolated myocytes[20], so it is clear that AE3 could, in principle, mediate recovery from an alkaline load. Numerous studies have shown that Cl^-/HCO_3^- exchange[25,26], and AE3 activity specifically[19,20], can mediate recovery from an experimentally-applied alkaline load in isolated cardiac myocytes. However, given the high rate of metabolism in the intact heart, with the accompanying generation of acid, primarily from CO_2 hydration[27], it is difficult to envision physiological conditions *in vivo* that would require a major capacity for recovery from an alkaline load. For example, it would not be credible to propose that the heart failure phenotype[24] or the impaired force-frequency response of AE3-null mice during pacing[23] might be due to an enhanced cytosolic alkaline load, particularly given that most of the HCO_3^- is derived from hydration of CO_2, with equimolar production of H^+. Nevertheless, the loss of AE3 could have a major effect on subsarcolemmal pH_i and, as discussed previously[23], affect biomechanical stress sensing and signaling at costameres, localized Na^+- and Ca^{2+}-loading, ion channel activities, or cytoskeletal function. It is therefore possible that effects on localized pH_i or secondary effects on intracellular Cl^- may underlie some of the functional deficits.

Expression changes in genes involved in membrane excitability and cardiac conduction. Gene expression changes provided strong evidence of electrical remodeling in AE3-null hearts (Fig. 4, see Supplementary Table S5 online for a listing of all genes for this section). These included altered expression of genes encoding a large number of cation channels. Among the voltage-sensitive sodium channels, **Scn5a** ($Na_v1.5$) and **Scn10a** ($Na_v1.8$) were upregulated and **Scn1b** and **Scn4b** regulatory beta subunits were downregulated. Scn5a is the primary Na^+ channel responsible for depolarization in atria, ventricles, and the conduction system[28]. Scn10a, expressed at much lower levels, has been reported in the conduction system[28]. Among voltage-sensitive Ca^{2+} channels, there was no change in expression of Cacna1c ($Ca_v1.2$; L-type α1C), the major Ca^{2+} channel in heart (RPKM = 45 in both genotypes). **Cacna2d2** (L-type $\alpha2/\delta2$ auxiliary subunit), **Cacna1d** ($Ca_v1.3$; L-type α1d), and **Cacna1g** ($Ca_v3.1$; T-type α1G) were upregulated. Cacna2d2 is expressed in atria[29], and Cacna1d and Cacna1g are expressed in pacemaking cells[30,31]. **Cacna1s** ($Ca_v1.1$; L-type α1S), the major skeletal muscle Ca^{2+} channel, was present at relatively low levels and was downregulated.

Most of the affected K^+ channel genes were upregulated. In addition to the K_{ATP} channels (Abcc8 and Abcc9) discussed in the section on metabolism, these included **Hcn4**, a hyperpolarization-activated cyclic nucleotide-gated K^+ channel expressed in the sinoatrial node[32]; **Kcnd2** ($K_v4.2$; a member of the Response to Hypoxia GO category) and **Kcna4** ($K_v1.4$), which mediate transient outward currents during the initial phase of depolarization[28]; **Kcnh2** (Kv11.1, ERG1), which is a major long QT syndrome gene; **Kcnj3** and **Kcnj5**, which are activated by acetylcholine and affect the atrial action potential and pacing[28,33]; and **Kcnn2**, a Ca^{2+}-activated K^+ channel that functions in the atrioventricular node[34]. The modulatory subunit **Kcne4** can associate with Kcnq1 ($K_v7.1$, 1.13-fold increase, $p = 0.023$) and inhibit its activity[35],

suggesting that reduced expression could increase Kcnq1 activity in the later phase of repolarization. **Kcne1** (minK), a modulatory subunit that associates with Kcnq1 and is a member of the cardiac conduction GO category, was upregulated; loss or mutations in Kcne1 leads to pacing-induced ventricular arrhythmias or long QT syndrome[36]. **Kcnj4** (K_{ir}2.3), an inwardly rectifying channel that operates in the final phases of repolarization and helps set the resting membrane potential[28], was the only major K^+ channel that was downregulated.

Additional upregulated genes with prominent functions in electrical excitability and conduction were **Gja1, Gja5, Dsc2, Dsg2, Hopx, and Popdc2**. All were members of one or more GO categories involved in cardiac conduction (Table 1). Gja1 and Gja5 (connexins 43 and 40) are gap junction proteins that function in electrical coupling for propagation of the depolarizing current[37]. Dsc2 (desmocollin 2) and Dsg2 (desmoglein 2) are components of the cell junction; they are required for mechanical coupling of cardiac myocytes, and particularly those of the conduction system, where mutations in desmosomal proteins are known to cause arrhythmias[38]. **Hopx** is a transcription factor that is essential for the development and maintenance of the conduction system[39]. **Popdc2** is a popeye domain-containing protein that plays a critical role in the pacemaking and conduction system, possibly by increasing expression of certain K^+ channels at the sarcolemma[40].

In addition to genes for channel proteins discussed above, Supplementary Table S6 online includes all of the transporters, pumps, and channels that were significantly changed. Some have the potential to affect conduction and electrical properties, as well as Na^+-loading and Ca^{2+}-loading discussed in the text. For example, Na/Ca/K exchangers of the Slc24a family are expressed at relatively low levels but their combined expression is ~20% of the levels of the NCX1 Na/Ca exchanger (Slc8a1) and both **Slc24a2** and **Slc24a4** were upregulated (1.51- and 2.0-fold). Although many of these genes, including that of the Na^+/K^+-ATPase α4 subunit (**Atp1a4**, 0.83-fold, Fig. 6) are expressed at low levels and cardiac expression has not been shown previously, if they were expressed in conductive tissue, they could play important roles.

The above results suggest that AE3-null cardiac myocytes have altered electrical properties and that conductive tissue is particularly affected. It is known that many ion channels are affected by hypoxia[41,42]. Furthermore, hypoxia has a strong effect on the action potential and is known to cause atrial fibrillation[43]. Thus, some of the changes in mRNA expression may represent an adaptive response to mild hypoxia resulting from impaired CO_2 disposal. On the other hand, it is also possible that some of these changes represent a response to perturbations of intracellular ion (HCO_3^- and Cl^-) and pH homeostasis caused by the loss of AE3.

Expression changes in genes encoding sarcomere and sarcomeric cytoskeletal proteins. Differentially expressed genes encoding myofibrillar proteins localized to the sarcomere, M-band, Z-discs, t-tubules, and intercalated discs are shown in Fig. 5 (see Supplementary Table 7 online for the full gene set). Among the myosin chains[44], the atrial myosin essential and regulatory light chains (**Myl4** and **Myl7**) were upregulated, but the corresponding ventricular light chains (Myl3 and Myl2) were not changed, suggesting that the atria were more affected than the ventricles by loss of AE3. **Myl12a** and **Myl12b** were both down-regulated. They are thought to serve as non-muscle myosin regulatory light chains[45]; however, each isoform is expressed at about half the levels of the atrial regulatory myosin light chain, suggesting an important function in heart. **Mybphl** (myosin binding protein H-like; 1.33-fold increase) has not been studied in heart, however, it is expressed at much higher levels than the closely related myosin binding protein H (Mybph), a skeletal muscle protein that has been identified in cardiac purkinje fibers[46]. The relatively high Mybphl levels and high similarity to the titin-binding

domain of cardiac myosin binding protein C suggest that it is likely to be a titin-binding protein and play a significant role in heart. **Myl1**, the skeletal muscle myosin essential light chain, was sharply downregulated (0.22-fold) and **Acta1**, skeletal muscle actin, was also reduced (0.37-fold); although they are expressed at only low levels relative to the cardiac isoforms, their downregulation suggests significant remodeling of the myofibrils. In contrast to the myosin light chains, expression of cardiac myosin heavy chains was not changed.

A number of differentially expressed mRNAs encode proteins that localize to the M-band. These include **Myom2** (myomesin 2, 1.32-fold increase; Myom1 was not changed), which crosslinks myosin heavy chains[47], and both **Obscn** (obscurin) and **Obsl1** (obscurin-like 1)**,** which are downregulated and are targeted to the M-band by interactions with titin and myomesin[48]. Obscurin is necessary for localization of **Ank2** (Ankyrin B, 1.10-fold increase) to the M-band, where Ank2 helps organize costameres and subsarcolemma microtubules[49]. **Lrrc39** (Momasp, Myosin interacting, M-band-associated stress-responsive protein; 1.21-fold increase) interacts with myosin heavy chains, is localized to the M-band, and regulates gene expression in response to biomechanical stress[50]. These M-band proteins may function in mechanical stress sensing[51] and modulation of passive force by titin.

mRNAs for some Z-disc proteins were also differentially expressed. **Myot** (myotilin), a Z-disc protein that can localize to the M-band under some conditions, was sharply downregulated. It has been suggested to function in myofibrillar assembly[52], although targeted deletion of Myot had no effect on contractility or muscle mass, possibly due to compensation by Tcap and other Z-disc proteins[53]. **Tcap** (titin-cap, 1.41-fold increase) is a Z-disc, titin-interacting protein that, among other functions, links t-tubules and myofibrils through its interaction with Kcne1[54], a K^+ channel β subunit discussed above. **Nebl** (nebulette), the smaller cardiac isoform of nebulin that interacts with various myofibrillar proteins, was upregulated. Interaction of Nebl with tropomyosin is important for thin filament stability in cardiac muscle[55]. **Mypn** (myopalladin) a multifunctional protein with many interacting partners, was downregulated. Interaction of Mypn with nebulette is necessary for Z-disc assembly, and it's interactions with CARP (cardiac ankyrin repeat protein, Ankrd1) regulate muscle gene expression[56]. **Palld** (palladin), which is related to myotilin and myopalladin, was upregulated; it binds actin and α-actinin, and is expressed in Z-discs, focal adhesions, and other structures, where it may function as a molecular scaffold[57]. **Lims2 (**Pinch2, 0.78-fold decrease) localizes to Z-discs and focal adhesions[58] and appears to be important in organization of intercalated discs and costameres and in signaling through integrin linked kinase and Akt[59]. **Xirp2**, which was downregulated, interacts with nebulette in the mature Z-disc and in developing myofibrils, and was proposed to function in myofibril development and remodeling[60]. Targeted deletion of Xirp2 also leads to defects in the development and maturation of intercalated discs[61].

Jph2 (junctophilin 2, 0.87-fold decrease) is a structural protein that connects the t-tubule to the sarcoplasmic reticulum by positioning the L-type calcium channel and ryanodine receptor. Cardiac specific knockdown of Jph2 in mice resulted in development of heart failure and reduced Ca^{2+}-induced Ca^{2+} release from the SR by regulating Ryr2[62]. Ankyrin B, discussed above, also plays an important role in positioning of transporters and channels[63].

Fhl1 and **Fhl2** (four-and-a half LIM-only proteins 1 and 2; both decreased) and **Ankrd23** (DARP, diabetes-related ankyrin-repeat protein; 0.65-fold decrease) interact with titin, have been proposed to be involved in stretch sensing, and all 3 proteins can relocalize to the nucleus to affect transcription[64]. Fhl1 localizes to both the Z-disc/I-band and M-band regions, interacts with myosin-binding protein C, and regulates sarcomere assembly[65]. Fhl2 also localizes to the I-band

and M-band and is involved in the recruitment of metabolic enzymes to titin[66]. When **Cryab** (αB-crystallin, 0.80-fold decrease) binds to titin in the I-band region, it increases passive tension[67], suggesting that its reduced expression may reduce tension.

Unlike some of the genes involved in hypoxia responses and metabolism, it is difficult to relate the changes in myofibrillar genes directly to deficits in CO_2 disposal and O_2 utilization. However, after translocation to the nucleus, both **Fhl1** and **Fhl2** inhibit Hif1a-mediated transcription[68,69], suggesting that reduced expression may increase transcription of Hif1a-regulated genes. **Sqstm1**, mentioned earlier for its role in hypoxia responses (Fig. 1), interacts with Nbr1 at the protein kinase domain of titin[70]. A possible function of the changes in some of the titin-binding proteins, including the reduction in αB-crystallin mentioned above[67], is to reduce passive tension. Phosphorylation of titin by PKA following β-adrenergic stimulation was shown to reduce passive tension[71] and the authors speculated that this allows more rapid and complete ventricular filling. A reduction in passive tension/stiffness may allow maintenance of appropriate cardiac output, despite reduced O_2 utilization.

Possible additional mechanisms for reducing passive tension/stiffness. Regarding passive tension/stiffness, mentioned above, some additional changes that could be adaptive in a heart with impaired O_2/CO_2 balance or other perturbations that affect contractility are downregulation of **Col4a1** (0.78-fold) and **Col4a2** (0.79-fold), two of the most abundantly expressed collagens in heart (Supplementary Table S1 online). Reduced expression of these basement membrane collagens, produced by cardiomyocytes[72], should reduce myocardial stiffness. Both are members of the Basement Membrane GO category (GO:0005604), which was significantly changed. Also downregulated were **Loxl1** (0.87-fold) and **Loxl2** (0.83-fold, also a member of GO:0005604), the most abundantly expressed lysyl oxidases in heart (Supplementary Table S1 online). Downregulation of lysyl oxidases, which can increase stiffness by crosslinking collagen, has the potential to reduce stiffness of the extracellular matrix[73].

Evidence that the differential mRNA expression changes in AE3-null hearts represent adaptive rather than pathological changes. A basic assumption in this study is that, in a mouse model that appears healthy under normal conditions, most of the mRNA expression changes will be compensatory, and can therefore yield insights into compensatory mechanisms that allow adaptation to physiological perturbations caused by a single gene deletion (e.g., the hypoxia response and altered energy metabolism genes in AE3-null hearts that may allow adaptation to impaired CO_2 disposal). In contrast, in a disease model, healthy adaptation is increasingly difficult and a major fraction of the gene expression changes may be non-adaptive or secondary to the disease process and reflect a deterioration of organ function rather than compensatory adaptation to a specific perturbation. Of course, this is not to say that all of the expression changes in the AE3-null heart are adaptive or that all expression changes in a heart disease model are non-adaptive.

AE3-null mice at 4 months of age, as used in this study, appear indistinguishable from their wild-type littermates and it is only when subjected to atrial pacing or when the AE3-null mutation is combined with another mutation that a clear phenotype is apparent[22-24]. Even in the pacing studies, the phenotype was mild and the ~60% increase in Akt phosphorylation in hearts of AE3-null mice, which correlated well with the induction of mRNA for Akt-responsive proteins involved in glucose metabolism (noted in Fig. 3), seems likely to provide some compensation for the force-frequency defect[23].

To test the assumption that differential gene expression patterns in the AE3-null heart were generally adaptive rather than pathological, we compared expression levels of the top 620 named genes that were significantly changed (FDR $\leq$ 0.075; P $\leq$ 0.0025) in the AE3-null heart with 1364 named genes that were changed by at least 1.5-fold (up-regulated or down-regulated) in a mouse model of heart failure (HF), as determined by RNA Seq analysis[74]. When the two data sets were compared, 134 genes overlapped, so ~10% of the HF genes were represented in the top 620 named genes in the AE3 data set. However, when the direction of change was analyzed, only 40 genes (30%) were changed in the same direction and 94 genes (70%) were changed in the opposite direction. Similar results were observed if we compared the 142 genes illustrated in Figs. 1-6 with the HF genes; 42/142 genes (30%) overlapped with the Heart Failure genes, with 14 (33%) changing in the same direction and 28 (67%) changing in the opposite direction. In addition, the magnitudes of the changes were much greater in the Heart Failure model. This suggests that the changes in AE3-null hearts are largely adaptive rather than pathological.

References

1 Cooper, G. J., Occhipinti, R. & Boron, W. F. CrossTalk proposal: Physiological CO_2 exchange can depend on membrane channels. *J Physiol* **593**, 5025-5028, doi:10.1113/JP270059 (2015).

2 Hulikova, A., Vaughan-Jones, R. D., Niederer, S. A. & Swietach, P. CrossTalk opposing view: Physiological CO_2 exchange does not normally depend on membrane channels. *J Physiol* **593**, 5029-5032, doi:10.1113/JP270013 (2015).

3 Cooper, G. J., Occhipinti, R. & Boron, W. F. Rebuttal from Gordon J. Cooper, Rossana Occhipinti and Walter F. Boron. *J Physiol* **593**, 5033, doi:10.1113/JP271239 (2015).

4 Swietach, P., Vaughan-Jones, R. D., Hulikova, A. & Niederer, S. A. Rebuttal from Pawel Swietach, Richard D. Vaughan-Jones, Alzbeta Hulikova and Steven A. Niederer. *J Physiol* **593**, 5035, doi:10.1113/JP271240 (2015).

5 Gawenis, L. R. *et al.* Mice with a targeted disruption of the AE2 Cl^-/HCO_3^- exchanger are achlorhydric. *J Biol Chem* **279**, 30531-30539, doi:10.1074/jbc.M403779200 (2004).

6 Schultheis, P. J. *et al.* Renal and intestinal absorptive defects in mice lacking the NHE3 Na^+/H^+ exchanger. *Nat Genet* **19**, 282-285, doi:10.1038/969 (1998).

7 Romero, M. F., Chen, A. P., Parker, M. D. & Boron, W. F. The SLC4 family of bicarbonate (HCO_3^-) transporters. *Mol Aspects Med* **34**, 159-182, doi:10.1016/j.mam.2012.10.008 (2013).

8 Cordat, E. & Casey, J. R. Bicarbonate transport in cell physiology and disease. *Biochem J* **417**, 423-439, doi:10.1042/BJ20081634 (2009).

9 Wang, H. S., Chen, Y., Vairamani, K. & Shull, G. E. Critical role of bicarbonate and bicarbonate transporters in cardiac function. *World J Biol Chem* **5**, 334-345, doi:10.4331/wjbc.v5.i3.334 (2014).

10 Newman, E. A. Sodium-bicarbonate cotransport in retinal Muller (glial) cells of the salamander. *J Neurosci* **11**, 3972-3983, doi:. (1991).

11 Kobayashi, S., Morgans, C. W., Casey, J. R. & Kopito, R. R. AE3 anion exchanger isoforms in the vertebrate retina: developmental regulation and differential expression in neurons and glia. *J Neurosci* **14**, 6266-6279, doi:. (1994).

12 Alvarez, B. V. *et al.* Blindness caused by deficiency in AE3 chloride/bicarbonate exchanger. *PLoS One* **2**, e839, doi:10.1371/journal.pone.0000839 (2007).

13 Casey, J. R., Sly, W. S., Shah, G. N. & Alvarez, B. V. Bicarbonate homeostasis in excitable tissues: role of AE3 Cl^-/HCO_3^- exchanger and carbonic anhydrase XIV interaction. *Am J Physiol Cell Physiol* **297**, C1091-1102, doi:10.1152/ajpcell.00177.2009 (2009).

14 Schroeder, M. A. *et al.* Extramitochondrial domain rich in carbonic anhydrase activity improves myocardial energetics. *Proc Natl Acad Sci U S A* **110**, E958-967, doi:10.1073/pnas.1213471110 (2013).

15 Barth, E., Stammler, G., Speiser, B. & Schaper, J. Ultrastructural quantitation of mitochondria and myofilaments in cardiac muscle from 10 different animal species including man. *J Mol Cell Cardiol* **24**, 669-681 (1992).

16 Vargas, L. A. & Alvarez, B. V. Carbonic anhydrase XIV in the normal and hypertrophic myocardium. *J Mol Cell Cardiol* **52**, 741-752, doi:10.1016/j.yjmcc.2011.12.008 (2012).

17 Garciarena, C. D., Youm, J. B., Swietach, P. & Vaughan-Jones, R. D. H^+-activated Na^+ influx in the ventricular myocyte couples Ca^{2+}-signalling to intracellular pH. *J Mol Cell Cardiol* **61**, 51-59, doi:10.1016/j.yjmcc.2013.04.008 (2013).

18 Alvarez, B. V., Fujinaga, J. & Casey, J. R. Molecular basis for angiotensin II-induced increase of chloride/bicarbonate exchange in the myocardium. *Circ Res* **89**, 1246-1253, doi:. (2001).

19 Chiappe de Cingolani, G. E. *et al.* Involvement of AE3 isoform of Na^+-independent Cl^-/HCO_3^- exchanger in myocardial pH_i recovery from intracellular alkalization. *Life Sci* **78**, 3018-3026, doi:10.1016/j.lfs.2005.11.030 (2006).

20 Sowah, D., Brown, B. F., Quon, A., Alvarez, B. V. & Casey, J. R. Resistance to cardiomyocyte hypertrophy in $ae3^{-/-}$ mice, deficient in the AE3 Cl^-/HCO_3^- exchanger. *BMC Cardiovasc Disord* **14**, 89, doi:10.1186/1471-2261-14-89 (2014).

21 Alvarez, B. V. *et al.* Carbonic anhydrase inhibition prevents and reverts cardiomyocyte hypertrophy. *J Physiol* **579**, 127-145, doi:10.1113/jphysiol.2006.123638 (2007).

22 Prasad, V. *et al.* Impaired cardiac contractility in mice lacking both the AE3 Cl^-/HCO_3^- exchanger and the NKCC1 Na^+-K^+-$2Cl^-$ cotransporter: effects on Ca^{2+} handling and protein phosphatases. *J Biol Chem* **283**, 31303-31314, doi:10.1074/jbc.M803706200 (2008).

23 Prasad, V. *et al.* Loss of the AE3 Cl^-/HCO_3^- exchanger in mice affects rate-dependent inotropy and stress-related AKT signaling in heart. *Front Physiol* **4**, 399, doi:10.3389/fphys.2013.00399 (2013).

24 Al Moamen, N. J. *et al.* Loss of the AE3 anion exchanger in a hypertrophic cardiomyopathy model causes rapid decompensation and heart failure. *J Mol Cell Cardiol* **50**, 137-146, doi:10.1016/j.yjmcc.2010.10.028 (2011).

25 Camilion de Hurtado, M. C., Alvarez, B. V., Perez, N. G., Ennis, I. L. & Cingolani, H. E. Angiotensin II activates Na^+-independent Cl^--HCO_3^- exchange in ventricular myocardium. *Circ Res* **82**, 473-481 (1998).

26 Xu, P. & Spitzer, K. W. Na-independent Cl^--HCO_3^- exchange mediates recovery of pHi from alkalosis in guinea pig ventricular myocytes. *Am J Physiol* **267**, H85-91, doi:. (1994).

27 Rose, B. D. *Clinical physiology of acid-base and electrolyte disorders*. 4th edn, (McGraw-Hill, Health Professions Division, 1994).

28 Schmitt, N., Grunnet, M. & Olesen, S. P. Cardiac potassium channel subtypes: new roles in repolarization and arrhythmia. *Physiol Rev* **94**, 609-653, doi:10.1152/physrev.00022.2013 (2014).

29 Hatano, S. *et al.* Molecular and electrophysiological differences in the L-type Ca^{2+} channel of the atrium and ventricle of rat hearts. *Circ J* **70**, 610-614, doi:. (2006).

30 Zhang, Z. *et al.* Functional Roles of $Ca_v1.3$ (α_{1D}) calcium channel in sinoatrial nodes: insight gained using gene-targeted null mutant mice. *Circ Res* **90**, 981-987, doi:. (2002).

31 Mesirca, P., Torrente, A. G. & Mangoni, M. E. T-type channels in the sino-atrial and atrioventricular pacemaker mechanism. *Pflugers Arch* **466**, 791-799, doi:10.1007/s00424-014-1482-6 (2014).

32 Baruscotti, M., Barbuti, A. & Bucchi, A. The cardiac pacemaker current. *J Mol Cell Cardiol* **48**, 55-64, doi:10.1016/j.yjmcc.2009.06.019 (2010).

33 Mesirca, P. *et al.* The G-protein-gated K^+ channel, IKACh, is required for regulation of pacemaker activity and recovery of resting heart rate after sympathetic stimulation. *J Gen Physiol* **142**, 113-126, doi:10.1085/jgp.201310996 (2013).

34 Zhang, Q. *et al.* Functional roles of a Ca^{2+}-activated K^+ channel in atrioventricular nodes. *Circ Res* **102**, 465-471, doi:10.1161/CIRCRESAHA.107.161778 (2008).

35 Manderfield, L. J. & George, A. L., Jr. KCNE4 can co-associate with the I(Ks) (KCNQ1-KCNE1) channel complex. *FEBS J* **275**, 1336-1349, doi:10.1111/j.1742-4658.2008.06294.x (2008).
36 Balasubramaniam, R., Grace, A. A., Saumarez, R. C., Vandenberg, J. I. & Huang, C. L. Electrogram prolongation and nifedipine-suppressible ventricular arrhythmias in mice following targeted disruption of KCNE1. *J Physiol* **552**, 535-546, doi:10.1113/jphysiol.2003.048249 (2003).
37 Jansen, J. A., van Veen, T. A., de Bakker, J. M. & van Rijen, H. V. Cardiac connexins and impulse propagation. *J Mol Cell Cardiol* **48**, 76-82, doi:10.1016/j.yjmcc.2009.08.018 (2010).
38 Mezzano, V., Pellman, J. & Sheikh, F. Cell junctions in the specialized conduction system of the heart. *Cell Commun Adhes* **21**, 149-159, doi:10.3109/15419061.2014.905928 (2014).
39 Ismat, F. A. *et al.* Homeobox protein Hop functions in the adult cardiac conduction system. *Circ Res* **96**, 898-903, doi:10.1161/01.RES.0000163108.47258.f3 (2005).
40 Froese, A. *et al.* Popeye domain containing proteins are essential for stress-mediated modulation of cardiac pacemaking in mice. *J Clin Invest* **122**, 1119-1130, doi:10.1172/JCI59410 (2012).
41 Shimoda, L. A. & Polak, J. Hypoxia. 4. Hypoxia and ion channel function. *Am J Physiol Cell Physiol* **300**, C951-967, doi:10.1152/ajpcell.00512.2010 (2011).
42 Hool, L. C. Acute hypoxia differentially regulates K^+ channels. Implications with respect to cardiac arrhythmia. *Eur Biophys J* **34**, 369-376, doi:10.1007/s00249-005-0462-3 (2005).
43 Lu, Z. *et al.* Increase in vulnerability of atrial fibrillation in an acute intermittent hypoxia model: importance of autonomic imbalance. *Auton Neurosci* **177**, 148-153, doi:10.1016/j.autneu.2013.03.014 (2013).
44 England, J. & Loughna, S. Heavy and light roles: myosin in the morphogenesis of the heart. *Cell Mol Life Sci* **70**, 1221-1239, doi:10.1007/s00018-012-1131-1 (2013).
45 Park, I. *et al.* Myosin regulatory light chains are required to maintain the stability of myosin II and cellular integrity. *Biochem J* **434**, 171-180, doi:10.1042/BJ20101473 (2011).
46 Alyonycheva, T., Cohen-Gould, L., Siewert, C., Fischman, D. A. & Mikawa, T. Skeletal muscle-specific myosin binding protein-H is expressed in Purkinje fibers of the cardiac conduction system. *Circ Res* **80**, 665-672 (1997).
47 Sequeira, V., Nijenkamp, L. L., Regan, J. A. & van der Velden, J. The physiological role of cardiac cytoskeleton and its alterations in heart failure. *Biochim Biophys Acta* **1838**, 700-722, doi:10.1016/j.bbamem.2013.07.011 (2014).
48 Fukuzawa, A. *et al.* Interactions with titin and myomesin target obscurin and obscurin-like 1 to the M-band: implications for hereditary myopathies. *J Cell Sci* **121**, 1841-1851, doi:10.1242/jcs.028019 (2008).
49 Randazzo, D. *et al.* Obscurin is required for ankyrinB-dependent dystrophin localization and sarcolemma integrity. *J Cell Biol* **200**, 523-536, doi:10.1083/jcb.201205118 (2013).
50 Will, R. D. *et al.* Myomasp/LRRC39, a heart- and muscle-specific protein, is a novel component of the sarcomeric M-band and is involved in stretch sensing. *Circ Res* **107**, 1253-1264, doi:10.1161/CIRCRESAHA.110.222372 (2010).

51 Gautel, M. The sarcomeric cytoskeleton: who picks up the strain? *Curr Opin Cell Biol* **23**, 39-46, doi:10.1016/j.ceb.2010.12.001 (2011).

52 Carlsson, L., Yu, J. G., Moza, M., Carpen, O. & Thornell, L. E. Myotilin: a prominent marker of myofibrillar remodelling. *Neuromuscul Disord* **17**, 61-68, doi:10.1016/j.nmd.2006.09.007 (2007).

53 Ochala, J., Carpen, O. & Larsson, L. Maintenance of muscle mass, fiber size, and contractile function in mice lacking the Z-disc protein myotilin. *Ups J Med Sci* **114**, 235-241, doi:10.3109/03009730903276399 (2009).

54 Furukawa, T. *et al.* Specific interaction of the potassium channel beta-subunit minK with the sarcomeric protein T-cap suggests a T-tubule-myofibril linking system. *J Mol Biol* **313**, 775-784, doi:10.1006/jmbi.2001.5053 (2001).

55 Bonzo, J. R., Norris, A. A., Esham, M. & Moncman, C. L. The nebulette repeat domain is necessary for proper maintenance of tropomyosin with the cardiac sarcomere. *Exp Cell Res* **314**, 3519-3530, doi:10.1016/j.yexcr.2008.09.001 (2008).

56 Bang, M. L. *et al.* Myopalladin, a novel 145-kilodalton sarcomeric protein with multiple roles in Z-disc and I-band protein assemblies. *J Cell Biol* **153**, 413-427 (2001).

57 Goicoechea, S. M., Arneman, D. & Otey, C. A. The role of palladin in actin organization and cell motility. *Eur J Cell Biol* **87**, 517-525, doi:10.1016/j.ejcb.2008.01.010 (2008).

58 Braun, A. *et al.* PINCH2 is a new five LIM domain protein, homologous to PINCHand localized to focal adhesions. *Exp Cell Res* **284**, 239-250 (2003).

59 Liang, X. *et al.* Targeted ablation of PINCH1 and PINCH2 from murine myocardium results in dilated cardiomyopathy and early postnatal lethality. *Circulation* **120**, 568-576, doi:10.1161/CIRCULATIONAHA.109.864686 (2009).

60 Eulitz, S. *et al.* Identification of Xin-repeat proteins as novel ligands of the SH3 domains of nebulin and nebulette and analysis of their interaction during myofibril formation and remodeling. *Mol Biol Cell* **24**, 3215-3226, doi:10.1091/mbc.E13-04-0202 (2013).

61 Wang, Q., Lin, J. L., Chan, S. Y. & Lin, J. J. The Xin repeat-containing protein, mXinbeta, initiates the maturation of the intercalated discs during postnatal heart development. *Dev Biol* **374**, 264-280, doi:10.1016/j.ydbio.2012.12.007 (2013).

62 van Oort, R. J. *et al.* Disrupted junctional membrane complexes and hyperactive ryanodine receptors after acute junctophilin knockdown in mice. *Circulation* **123**, 979-988, doi:10.1161/CIRCULATIONAHA.110.006437 (2011).

63 Hund, T. J. *et al.* Regulation of the ankyrin-B-based targeting pathway following myocardial infarction. *Cardiovasc Res* **81**, 742-749, doi:10.1093/cvr/cvn348 (2009).

64 Anderson, B. R. & Granzier, H. L. Titin-based tension in the cardiac sarcomere: molecular origin and physiological adaptations. *Prog Biophys Mol Biol* **110**, 204-217, doi:10.1016/j.pbiomolbio.2012.08.003 (2012).

65 McGrath, M. J. *et al.* Four and a half LIM protein 1 binds myosin-binding protein C and regulates myosin filament formation and sarcomere assembly. *J Biol Chem* **281**, 7666-7683, doi:10.1074/jbc.M512552200 (2006).

66 Lange, S. *et al.* Subcellular targeting of metabolic enzymes to titin in heart muscle may be mediated by DRAL/FHL-2. *J Cell Sci* **115**, 4925-4936, doi:. (2002).

67 Bullard, B. *et al.* Association of the chaperone αB-crystallin with titin in heart muscle. *J Biol Chem* **279**, 7917-7924, doi:10.1074/jbc.M307473200 (2004).

68 Hubbi, M. E., Gilkes, D. M., Baek, J. H. & Semenza, G. L. Four-and-a-half LIM domain proteins inhibit transactivation by hypoxia-inducible factor 1. *J Biol Chem* **287**, 6139-6149, doi:10.1074/jbc.M111.278630 (2012).
69 Lin, J. *et al.* FHL family members suppress vascular endothelial growth factor expression through blockade of dimerization of HIF1α and HIF1β. *IUBMB Life* **64**, 921-930, doi:10.1002/iub.1089 (2012).
70 Lange, S. *et al.* The kinase domain of titin controls muscle gene expression and protein turnover. *Science* **308**, 1599-1603, doi:10.1126/science.1110463 (2005).
71 Yamasaki, R. *et al.* Protein kinase A phosphorylates titin's cardiac-specific N2B domain and reduces passive tension in rat cardiac myocytes. *Circ Res* **90**, 1181-1188, doi:. (2002).
72 Lundgren, E. *et al.* In vitro studies on adult cardiac myocytes: attachment and biosynthesis of collagen type IV and laminin. *J Cell Physiol* **136**, 43-53, doi:10.1002/jcp.1041360106 (1988).
73 Gonzalez-Santamaria, J. *et al.* Matrix cross-linking lysyl oxidases are induced in response to myocardial infarction and promote cardiac dysfunction. *Cardiovasc Res* **109**, 67-78, doi:10.1093/cvr/cvv214 (2016).
74 Lee, J. H. *et al.* Analysis of transcriptome complexity through RNA sequencing in normal and failing murine hearts. *Circ Res* **109**, 1332-1341, doi:10.1161/CIRCRESAHA.111.249433 (2011).
75 Eden, E., Navon, R., Steinfeld, I., Lipson, D. & Yakhini, Z. GOrilla: a tool for discovery and visualization of enriched GO terms in ranked gene lists. *BMC Bioinformatics* **10**, 48, doi:10.1186/1471-2105-10-48 (2009).
76 Becker, K. G. *et al.* PubMatrix: a tool for multiplex literature mining. *BMC Bioinformatics* **4**, 61, doi:10.1186/1471-2105-4-61 (2003).
77 Svichar, N. *et al.* Carbonic anhydrases CA4 and CA14 both enhance AE3-mediated Cl^--HCO_3^- exchange in hippocampal neurons. *J Neurosci* **29**, 3252-3258, doi:10.1523/JNEUROSCI.0036-09.2009 (2009).
78 Jentsch, T. J. CLC chloride channels and transporters: from genes to protein structure, pathology and physiology. *Crit Rev Biochem Mol Biol* **43**, 3-36, doi:10.1080/10409230701829110 (2008).
79 Tian, Y., Schreiber, R. & Kunzelmann, K. Anoctamins are a family of Ca^{2+}-activated Cl^- channels. *J Cell Sci* **125**, 4991-4998, doi:10.1242/jcs.109553 (2012).
80 Kunzelmann, K. *et al.* Role of the Ca^{2+} -activated Cl^- channels bestrophin and anoctamin in epithelial cells. *Biol Chem* **392**, 125-134, doi:10.1515/BC.2011.010 (2011).
81 Voss, F. K. *et al.* Identification of LRRC8 heteromers as an essential component of the volume-regulated anion channel VRAC. *Science* **344**, 634-638, doi:10.1126/science.1252826 (2014).
82 Qiu, Z. *et al.* SWELL1, a plasma membrane protein, is an essential component of volume-regulated anion channel. *Cell* **157**, 447-458, doi:10.1016/j.cell.2014.03.024 (2014).
83 Yu, Y. *et al.* A rat RNA-Seq transcriptomic BodyMap across 11 organs and 4 developmental stages. *Nat Commun* **5**, 3230, doi:10.1038/ncomms4230 (2014).
84 Brawand, D. *et al.* The evolution of gene expression levels in mammalian organs. *Nature* **478**, 343-348, doi:10.1038/nature10532 (2011).

Supplemental Figures and Tables

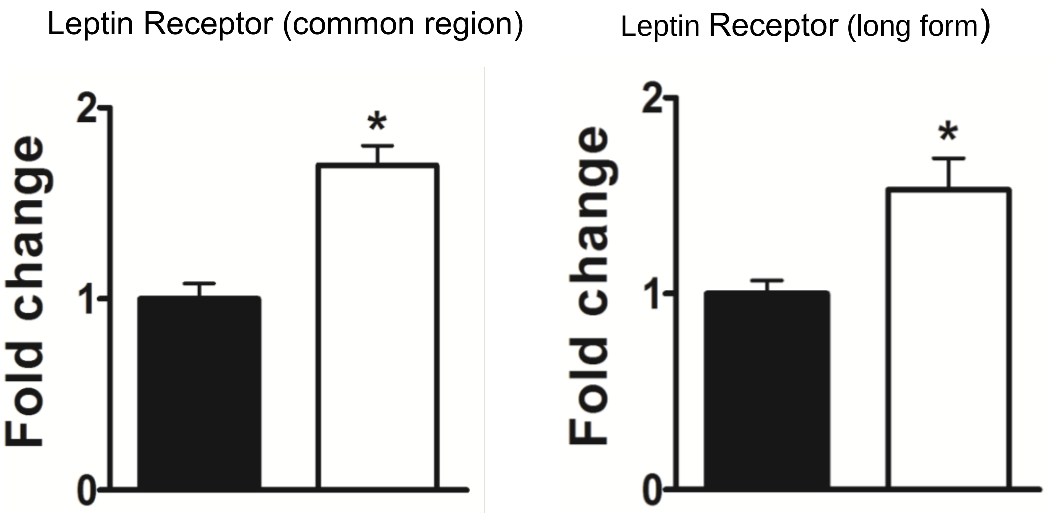

Supplementary Figure S1. RT-PCR analysis of Leptin Receptor mRNA. The Leptin Receptor includes multiple short forms with truncated C-termini and a long form (ObRb) with an extensive cytoplasmic C-terminal region. To determine whether the observed up-regulation (see Fig. 3) included the long-form, which contains the major signaling domains, quantitative RT-PCR of heart mRNA (n = 4 mice of each genotype) was performed. The following primers were used: Common form: Forward primer, GTCTTCGGGGATGTGAATGTC and Reverse primer, ACCTAAGGGTGGATCGGGTTT; unique regions present in the C-terminal domains of the long form: Forward primer, TGGTCCCAGCAGCTATGGT and Reverse primer, ACCCAGAGAAGTTAGCACTGT. Fold-change expression levels for AE3-null (white bars) vs WT (black bars) are shown. The common regions (1.71 ± 0.10 increase; P = 0.002) and long-form regions (1.54 ± 0.16 increase; P = 0.022) exhibited similar increases in AE3-null hearts. Amplification occurred at a higher cycle number for the long form, suggesting that it is a lower abundance splice variant in mouse heart.

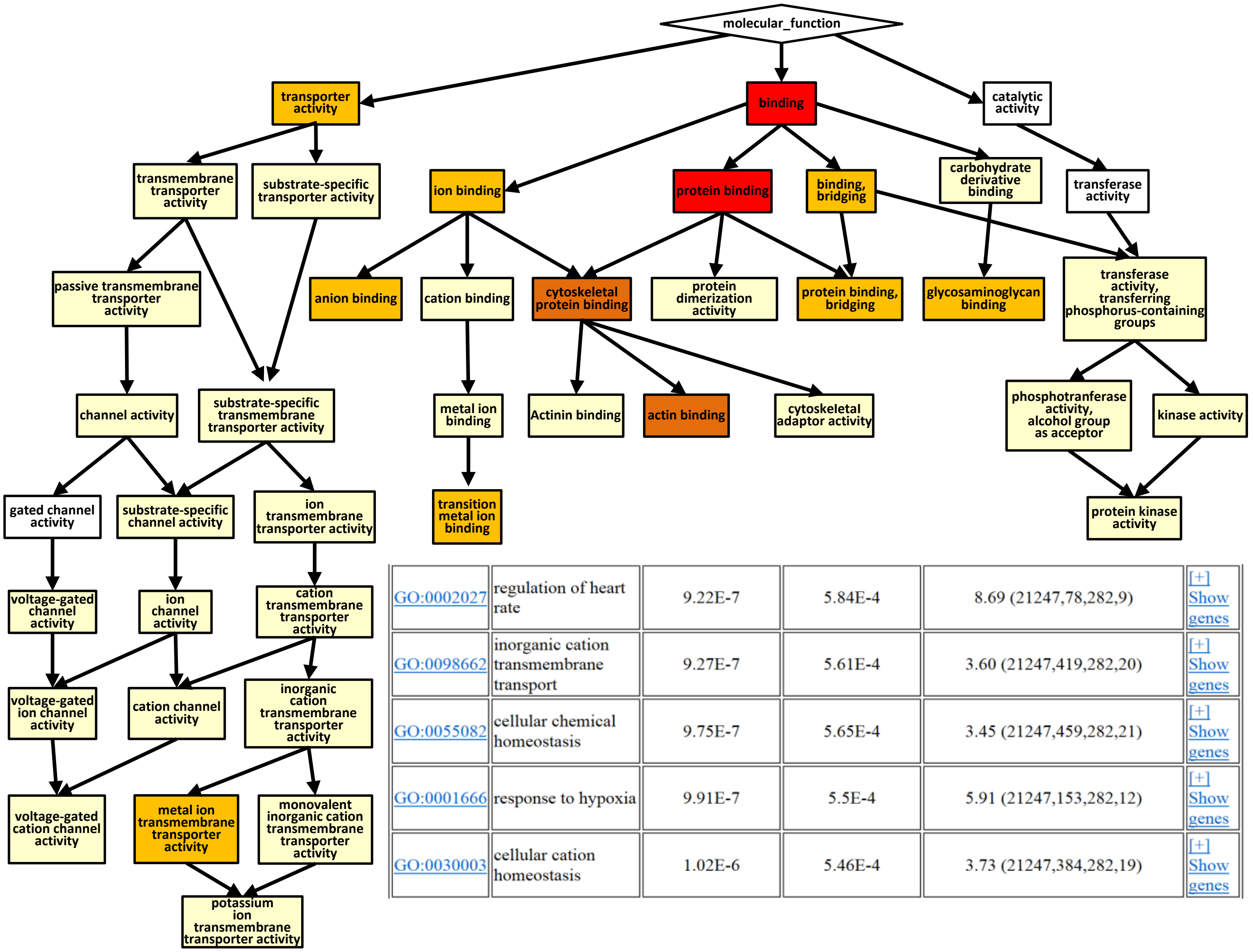

GO:0002027	regulation of heart rate	9.22E-7	5.84E-4	8.69 (21247,78,282,9)	[+] Show genes
GO:0098662	inorganic cation transmembrane transport	9.27E-7	5.61E-4	3.60 (21247,419,282,20)	[+] Show genes
GO:0055082	cellular chemical homeostasis	9.75E-7	5.65E-4	3.45 (21247,459,282,21)	[+] Show genes
GO:0001666	response to hypoxia	9.91E-7	5.5E-4	5.91 (21247,153,282,12)	[+] Show genes
GO:0030003	cellular cation homeostasis	1.02E-6	5.46E-4	3.73 (21247,384,282,19)	[+] Show genes

Supplementary Figure S2. Representative output from GOrilla Gene Ontology (GO) program (Gene Ontology enRIchment anaLysis and visuaLizAtion tool; http://cbl-gorilla.cs.technion.ac.il/)[75]. The diagram illustrates some of the nested GO Molecular Function categories obtained during analysis of genes that were significantly changed in AE3-null hearts. The user can switch between Biological Function, Molecular Function, and Cellular Component GO subcategories. Color-coding indicates degree of significance (red > dark orange > light orange > yellow). In addition to the visual display, a listing of all significant GO categories is provided with an option to Show genes or Hide genes (provided as gene symbols and descriptions); in the analysis shown, genes with FDR < 0.01 were used to examine Biological Process GO categories. The first P value listed is a raw P value and the second P value is an adjusted P value (or False Discovery Rate) that takes into consideration the large number of total GO categories, thereby reducing false positive GO categories. The final column gives the enrichment score and the numbers of genes (N, B, n, b) on which the enrichment was based (see Gene Ontology Analysis in Methods).

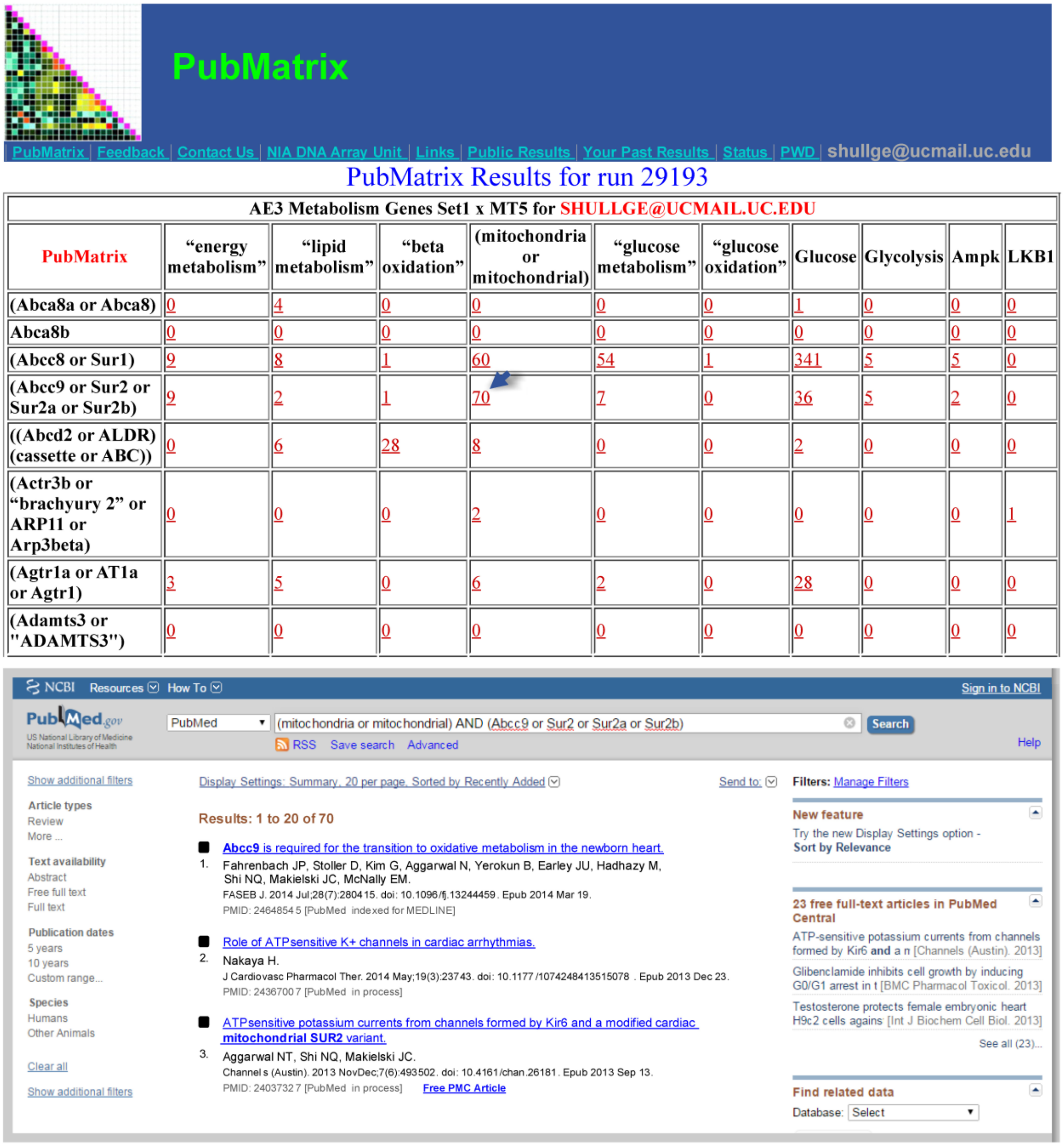

AE3 Metabolism Genes Set1 x MT5 for SHULLGE@UCMAIL.UC.EDU										
PubMatrix	"energy metabolism"	"lipid metabolism"	"beta oxidation"	(mitochondria or mitochondrial)	"glucose metabolism"	"glucose oxidation"	Glucose	Glycolysis	Ampk	LKB1
(Abca8a or Abca8)	0	4	0	0	0	0	1	0	0	0
Abca8b	0	0	0	0	0	0	0	0	0	0
(Abcc8 or Sur1)	9	8	1	60	54	1	341	5	5	0
(Abcc9 or Sur2 or Sur2a or Sur2b)	9	2	1	70	7	0	36	5	2	0
((Abcd2 or ALDR) (cassette or ABC))	0	6	28	8	0	0	2	0	0	0
(Actr3b or "brachyury 2" or ARP11 or Arp3beta)	0	0	0	2	0	0	0	0	0	1
(Agtr1a or AT1a or Agtr1)	3	5	0	6	2	0	28	0	0	0
(Adamts3 or "ADAMTS3")	0	0	0	0	0	0	0	0	0	0

Supplementary Figure S3. Representative output from PubMatrix literature searches[76]. Significantly changed genes (535 genes, FDR < 0.05) were run against modifier terms for various aspects of energy metabolism as shown in the top panel. Each set of searches is displayed on a grid that allows any pairwise search to be opened. In the example shown (upper panel), Abcc9, also known as Sur2, had 70 literature hits when searched against "(mitochondria or mitochondrial)". When a given search is opened, a listing of papers is provided by PubMed, a resource of the U.S. National Library of Medicine (bottom panel). The reader can access the searches performed here for Hypoxia (8 searches) and Metabolism (8 searches) genes by registering on PubMatrix with a username (typically email address) and password. The searches are archived under Public Results, username Vair&Shull. Searches were kindly transferred to Public Results by Dr. Kevin G. Becker of the National Institutes of Health.

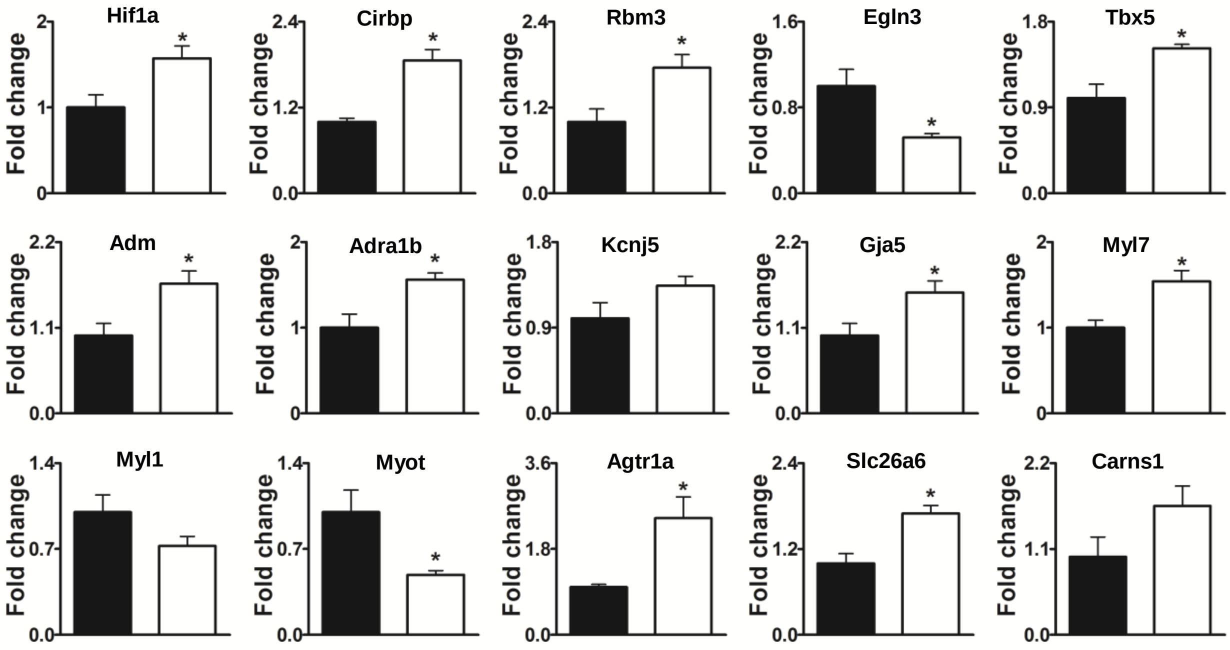

Supplementary Figure S4. Validation of RNA Seq Data by Real-time PCR Analysis. Expression of selected genes that exhibited significant changes by RNA-Seq Analysis were analyzed by Quantitative RT-PCR and normalized by Gapdh expression. Among the 15 genes analyzed (n = 4 hearts of each genotype), all exhibited changes in the same direction as observed in RNA Seq analysis, and 12/15 genes exhibited *P values < 0.05. P values for remaining 3 genes were: Kcnj5, 0.122; Myl1, 0.135; and Carns1, 0.116). Variability for each gene was far less in the RNA Seq analysis than by RT-PCR, indicating a much greater degree of reliability in the direct measurements provided by RNA Seq analysis rather than the amplification procedure provided by RT-PCR.

Legends for Supplementary Tables S1-S7 online, which are provided as Excel Files

Supplementary Table S1 online. Differential gene expression data in FVBN AE3-null vs wild-type mouse whole hearts. This table presents expression levels of the 23,281 genes examined by RNA Seq analysis. Genes are identified by gene identification numbers, gene symbol and gene description (if known), or by Riken numbers or accession numbers if for a gene of unknown function. Columns contain individual RPKM values for 4 WT (WT1-4) and 4 AE3-null (KO1-4) heart samples (male, 4-months old); mean values, standard errors, and standard deviations for WT and AE3-null samples; fold-change; log2fold-change, p-value, and adjusted p-value or False Discovery Rate (FDR) that takes into account the overall number of genes examined. This table can be used to compare relative expression levels of various genes in male FVBN mouse hearts.

Supplementary Table S2 online. Differentially expressed genes involved in Hypoxia, Vasodilation, and Angiogenesis. Relevant genes were identified by analyzing all genes with P values $\leq$ 0.01 using the GOrilla Gene Ontology (GO) program and by PubMatrix analysis of genes with FDR < 0.05. See methods for those procedures and for explanation of P-values, enrichment scores, and gene numbers (N, B, n, b) in GO analysis. The following GO categories were identified: **GO:0001525** Angiogenesis, P = 8.08E-12, 3.57 enrichment, (21238,231,979,38); **GO:0045765** Regulation of Angiogenesis, P = 5.05E-9, 3.37 enrichment (21238,193,979,30); **GO:0001666** Response to Hypoxia, P = 7.5E-5, 2.71 enrichment (21238,152,979,19); **GO:0019229** Regulation of Vasoconstriction, P = 6.51E-4, 3.39 enrichment (21238,64,979,10); **GO:0042312** Regulation of Vasodilation, P = 7.86E-04, 3.96 enrichment, (21238,44,979,8). For PubMatrix analysis we searched all genes with FDR < 0.05 with the following Modifier Terms: (hypoxia or hypoxic), (HIF1 or Hif1alpha or Hif1a or Hif), (Egln3 or PHD3), (Epas or Hif2a), (Vegf or Vegfa), Angiogenesis, Vasodilation, and Vasoconstriction. Genes (143 total) identified in these GO categories or judged to have significant involvement in hypoxia, angiogenesis, or vasodilation based on the literature identified in Pubmatrix are listed in the Excel file.

Supplementary Table S3 online. Differentially expressed genes involved in Signaling. Relevant genes (158 total) were identified by analyzing all genes with P values $\leq$ 0.01 using the GOrilla Gene Ontology program. See methods for those procedures and for explanation of P-values, enrichment scores, and gene numbers (N, B, n, b) in GO analysis. The following GO categories were identified: **GO:0007169** Transmembrane Receptor Protein Tyrosine Kinase Signaling Pathway, P = 1E-6, 2.62 enrichment, (21258,254,991,31); **GO:0043122** Regulation of I-KappaB Kinase/NF-KappaB Signaling, P = 3.51E-4, 2.41 enrichment, (21258,169,991,19); **GO:0008286** Insulin Receptor Signaling Pathway, P = 3.83E-4, 3.94 enrichment, (21258,49,991,9); **GO:0008277** Regulation of G-Protein Coupled Receptor Protein Signaling Pathway, P = 4.57E-4, 2.94 enrichment, (21258,95,991,13); **GO:0070372** Regulation of ERK1 and ERK2 cascade, P = 5.07E-4, 2.48 enrichment, (21258,147,991,17); **GO:0032147** Activation of Protein Kinase Activity, P = 5.12E-4, 2.28 enrichment, (21258,188,991,20); **GO:0014066** Regulation of Phosphatidylinositol 3-Kinase Signaling, P = 8.05E-4, 3.30 enrichment, (21258,65,991,10); **GO:0034976** Response to Endoplasmic Reticulum Stress, P = 6.95E-4, 2.96 enrichment, (21258,87,991,12); **GO:0004672** Protein Kinase Activity, P = 6.18E-5, 1.80 enrichment, (21258,571,991,48); **GO:0035591** Signaling Adaptor Activity, P = 1.33E-4, 5.05

enrichment, (21258,34,991,8); **GO:001630**1 Kinase Activity, P = 1.47E-4, 1.64 enrichment, (21258,774,991,59); **GO:0051020** GTPase Binding, P = 1.65E-4, 2.42 enrichment, (21258,186,991,21); **GO:000507**0 SH3/SH2 Adaptor Activity, P = 2.23E-4, 6.44 enrichment, (21258,20,991,6); **GO:0004683** Calmodulin-Dependent Protein Kinase Activity, P = 6.6E-4, 5.36 enrichment, (21258,24,991,6); **GO:0004674** Protein Serine/Threonine Kinase Activity, P = 9.97E-4, 1.75 enrichment, (21258,429,991,35).

Supplementary Table S4 online. Differentially expressed genes involved in Energy Metabolism. Relevant genes were identified by analyzing all genes with P values $\leq$ 0.01 using the GOrilla Gene Ontology program and by PubMatrix analysis of genes with FDR $\leq$ 0.05. We also used the Single Rank GOrilla option, which gave a better understanding of the enrichment of carbohydrate metabolism genes among the most significantly changed genes. See methods for explanation of P-values, enrichment scores, and gene numbers (N, B, n, b). The following GO categories were identified: **GO:0006109** Regulation of Carbohydrate Metabolic Process, P = 1.41E-5, 2.95 enrichment (21238,147,979,20); **GO:0045913** Positive Regulation of Carbohydrate Metabolic Process, P = 2.97E-5, 14.96 enrichment (21257,52,164,6); **GO:1900077** Negative Regulation of Cellular Response to Insulin Stimulus, P = 3.56E-5, 8.06 enrichment (21257,30,703,8); **GO:0010906** Regulation of Glucose Metabolic Process, P = 6.16E-5, 4.60 enrichment (21257,99,607,13); **GO:0043470** Regulation of Carbohydrate Catabolic Process, P = 7.34E-5, 17.97 enrichment (21257,35,169,5); **GO:0008286** Insulin Receptor Signaling Pathway, P = 8.09E-5, 6.52 enrichment (21257,49,599,9); **GO:0019216** Regulation of Lipid Metabolic Process, P = 1.11E-4, 2.57 enrichment (21257,230,863,24); **GO:0006110** Regulation of Glycolytic Process, P = 3.48E-4, 19.35 enrichment (21257,26,169,4); **GO:0032869** Cellular Response to Insulin Stimulus, P = 3.96E-4, 3.80 enrichment (21257,91,800,13); **GO:0032868** Response to insulin, P = 4.36E-4, 3.84 enrichment (21257,120,599,13). For PubMatrix analysis we searched all genes with FDR < 0.05 with the following Modifier Terms: "energy metabolism", "lipid metabolism", "beta oxidation", (mitochondria or mitochondrial), "glucose metabolism", "glucose oxidation", Glucose, Glycolysis, Ampk, Akt. Genes (142 total) identified in these GO categories or judged to have significant involvement in energy metabolism based on the literature identified in Pubmatrix are listed in the Excel file.

Supplementary Table S5 online. Differentially expressed genes involved in membrane excitability and cardiac conduction. Relevant genes were identified by analyzing all genes with P values $\leq$ 0.01 using the GOrilla Gene Ontology program. See methods for explanation of P-values, enrichment scores, and gene numbers (N, B, n, b). The following GO categories were identified: **GO:0008016** Regulation of Heart Contraction, P = 3.76E-10, 4.22 enrichment (21228,132,990,26); **GO:0003254** Regulation of Membrane Depolarization 1.42E-8, 7.80 enrichment (21228,33,990,12); **GO:0002027** Regulation of Heart Rate, P = 1.55E-8, 5.21 enrichment (21228,70,990,17); **GO:0061337** Cardiac Conduction, P = 2.22E-8, 8.42 enrichment (21228,28,990,11); **GO:0086091** Regulation of Heart Rate by Cardiac Conduction, P = 1.66E-6, 8.58 enrichment (21228,20,990,8); **GO:0042391** Regulation of Membrane Potential, P = 2.31E-6, 2.52 enrichment (21228,264,990,31); **GO:0060307** Regulation of Ventricular Cardiac Muscle Cell Membrane Repolarization, P = 3.83E-6, 11.70 enrichment (21228,11,990,6); **GO:0060371** Regulation of Atrial Cardiac Muscle Cell Membrane Depolarization, P = 1.09E-5, 13.40 enrichment (21228,8,990,5); **GO:0086001** Cardiac Muscle Cell Action Potential, P = 1.16E-5, 6.86 enrichment (21228,25,990,8); **GO:0086065** Cell Communication Involved in Cardiac

Conduction, P = 2.2E-5, 9.19 enrichment (21228,14,990,6); **GO:0001508** Action Potential, P = 5.54E-5, 3.84 enrichment (21228,67,990,12); **GO:0051899** Membrane Depolarization, 1.59E-4, 3.09 enrichment (21228,97,990,14); **GO:0086069** Bundle of His cell to Purkinje Myocyte Communication, 2.83E-4, 10.72 enrichment (21228,8,990,4); **GO:0086010** Membrane Depolarization during action potential, P = 8.36E-4, 4.41 enrichment (21228,34,990,7); **GO:0086005** Ventricular Cardiac Muscle Cell Action Potential, P = 8.45E-4, 6.31 enrichment (21228,17,990,5); **GO:0086067** AV Node Cell to Bundle of His Cell Communication, P = 9.42E-4, 12.87 enrichment (21228,5,990,3).

In addition to the above GO categories, other GO categories also contain genes such as cation channels that are potentially relevant to cardiac conduction and membrane excitability, and are therefore included in the table. These are: **GO:0051924** Regulation of Calcium Ion Transport, P = 6.32E-6, 2.77 enrichment (21228,186,990,24); **GO:0006813** Potassium Ion Transport, P = 2.26E-5, 2.86 enrichment (21228,150,990,20); **GO:0098662** Inorganic Cation Transmembrane Transport, P = 2.35E-5, 2.05 enrichment (21228,397,990,38); **GO:0098655** Cation Transmembrane Transport, P = 5.23E-5, 1.90 enrichment (21228,473,990,42); **GO:0006816** Calcium Ion Transport, P = 8.73E-4, 2.19 enrichment (21228,196,990,20); **GO:0022843** Voltage-gated Cation Channel Activity, P = 5.03E-5, 2.79 enrichment (21228,146,990,19); **GO:0015079** Potassium Ion Transmembrane Transporter Activity, P = 5.16E-5, 2.88 enrichment (21228,134,990,18); **GO:0015267** Channel Activity, P = 7.27E-5, 1.99 enrichment (21228,388,990,36); **GO:0005244** Voltage-gated Ion Channel Activity, P = 1.32E-4, 2.46 enrichment (21228,183,990,21). Genes (104 total) identified in the above GO categories or judged to have significant involvement in Cardiac Conduction or Membrane Excitability based on literature searches are listed in the Excel file.

Supplementary Table S6 online. Differentially expressed genes encoding Transporters, Pumps, and Channels. Relevant genes (84 total) were identified by analyzing all genes with P values $\leq$ 0.017 (the top 1250 genes) using the GOrilla Gene Ontology program for significantly enriched transport-related GO categories and by inspection of the list for genes with designations (e.g., Slc, Kcn, Atp, Abc, etc) indicating that they were transporters, pumps, or channels. Known direct regulators of pumps or channels such as phospholamban, sarcolipin, and junctophilins were also included. See methods for explanation of P-values, enrichment scores, and gene numbers (N, B, n, b). The following GO categories were identified: **GO:0030001** Metal Ion Transport, P = 6.97E-10, 3.14 enrichment, (21257,512,556,42); **GO:0006812** Cation Transport, P = 4.47E-9, 3.80 enrichment, (21257,680,247,30); **GO:0043269** Regulation of Ion Transport, P = 7.5E-9, 2.99 enrichment, (21257,485,586,40); **GO:0034765** Regulation of Ion Transmembrane Transport, P = 3.47E-8, 5.57 enrichment, (21257,278,247,18); **GO:0006811** Ion Transport, P = 1.1E-7, 2.21 enrichment, (21257,1004,564,59); **GO:0006873** Cellular Ion Homeostasis, P = 1.67E-7, 3.06 enrichment, (21257,348,639,32); **GO:0034220** Ion Transmembrane Transport, P = 2.5E-7, 3.78 enrichment, (21257,546,247,24); **GO:0030003** Cellular Cation Homeostasis, P = 2.55E-7, 3.07 enrichment, (21257,336,639,31); **GO:0015672** Monovalent Inorganic Cation Transport, P = 2.63E-7, 4.87 enrichment, (21257,318,247,18); **GO:0055085** Transmembrane Transport, P = 3.12E-7, 3.23 enrichment, (21257,772,247,29); **GO:0055080** Cation Homeostasis, P = 6.43E-7, 2.90 enrichment, (21257,409,573,32); **GO:0050801** Ion Homeostasis, P = 1.3E-6, 2.76 enrichment, (21257,444,573,33); **GO:0051924** Regulation of Calcium Ion Transport, P = 1.11E-5, 3.22 enrichment, (21257,162,857,21); **GO:0055065** Metal Ion Homeostasis, P = 1.33E-5, 2.92 enrichment, (21257,365,499,25); **GO:0046916** Cellular

Transition Metal Ion Homeostasis, P = 2.57E-5, 7.58 enrichment, (21257,53,476,9); **GO:0006813** Potassium Ion Transport, P = 3.11E-5, 4.17 enrichment, (21257,149,479,14); **GO:0030004** Cellular Monovalent Inorganic Cation Homeostasis, P = 1.1E-4, 7.24 enrichment, (21257,41,573,8); **GO:0006816** Calcium Ion Transport, P = 2.06E-4, 3.18 enrichment, (21257,194,551,16); **GO:0035725** Sodium Ion Transmembrane Transport, P = 2.86E-4, 3.57 enrichment, (21257,70,1190,14); **GO:0071804** Cellular Potassium Ion Transport, P = 2.92E-4, 3.76 enrichment, (21257,122,556,12); **GO:0071805** Potassium Ion Transmembrane Transport, P = 9.8E-4, 2.88 enrichment, (21257,121,1036,17).

Supplementary Table S7 online. Differentially expressed genes involved in the Sarcomere and Sarcomeric Cytoskeleton. Relevant genes were identified by analyzing all genes with P values $\leq$ 0.01 using the GOrilla Gene Ontology program. See methods for those procedures and for explanation of P-values, enrichment scores, and gene numbers (N, B, n, b) in GO analysis. The following GO categories were identified: **GO:0044449** Contractile Fiber Part, P = 2.93E-14, 4.57 enrichment (21238,166,979,35); **GO:0032432** Actin Filament Bundle, P = 3.06E-11, 6.54 enrichment (21238,63,979,19); **GO:0001725** Stress Fiber, P = 5.53E-10, 6.36 enrichment (21238,58,979,17); **GO:0042641** Actomyosin, P = 4.97E-9, 5.59 enrichment (21238,66,979,17); **GO:0030018** Z Disc, P = 8.18E-9, 4.62 enrichment (21238,94,979,20); **GO:0015629** Actin Cytoskeleton, P = 1.68E-7, 3.19 enrichment (21238,177,979,26); **GO:0030315** T-tubule, P = 2.19E-7, 5.76 enrichment (21238,49,979,13); **GO:0043292** Contractile Fiber, P = 6.03E-6, 4.73 enrichment (21238,55,979,12); **GO:0014704** Intercalated Disc, P = 3.71E-5, 4.72 enrichment (21238,46,979,10); **GO:0031674** I band, P = 2.82E-4, 6.20 enrichment (21238,21,979,6); **GO:0016459** Myosin Complex, P = 3.32E-4, 3.68 enrichment (21238,59,979,10); **GO:0003779** Actin Binding, P = 3.5E-12, 3.00 enrichment (21238,361,979,50); **GO:0051393** Alpha-Actinin Binding, P = 2.82E-4, 6.20 enrichment (21238,21,979,6); **GO:0031432** Titin Binding, P = 4.69E-4, 9.64 enrichment (21238,9,979,4); **GO:0007015** Actin Filament Organization, P = 4.45E-9, 3.79 enrichment (21238,149,979,26); **GO:0051494** Negative Regulation of Cytoskeleton Organization, P = 5.35E-7, 3.78 enrichment (21238,109,979,19); **GO:0008064** Regulation of Actin Polymerization or Depolymerization, P = 6.72E-6, 3.34 enrichment (21238,117,979,18); **GO:0051693** Actin Filament Capping, P = 2.77E-4, 5.24 enrichment (21238,29,979,7). Genes (116 total) identified in these GO categories are listed in the Excel file.

Supplementary Table S8. Expression of Carbonic Anhydrase mRNAs in Mouse Heart.

Gene	Description	WT Mean ± SE	KO Mean ± SE	Fold Change	P-value
Car1	Carbonic anhydrase 1	0	0	not exp	n/a
Car2	Carbonic anhydrase 2	3.47 ± 0.10	3.43 ± 0.17	0.97	0.99
Car3	Carbonic anhydrase 3	12.9 ± 1.2	15.0 ± 3.2	1.14	0.88
Car4	Carbonic anhydrase 4	10.6 ± 0.8	9.75 ± 0.61	0.91	0.28
Car5a	Carbonic anhydrase 5a	0.01 ± 0.01	0.03 ± 0.02	not exp	n/a
Car5b	Carbonic anhydrase 5b	0.40 ± 0.07	0.64 ± 0.02	1.60	0.01
Car6	Carbonic anhydrase 6	0	0.01 ± 0.01	not exp	n/a
Car7	Carbonic anhydrase 7	3.54 ± 0.50	4.42 ± 0.12	1.23	0.14
Car8	Carbonic anhydrase 8	3.16 ± 0.18	3.54 ± 0.21	1.10	0.12
Car9	Carbonic anhydrase 9	0.48 ± 0.06	0.46 ± 0.02	0.94	0.80
Car10	Carbonic anhydrase 10	0.01 ± 0.01	0.01 ± 0.01	not exp	n/a
Car11	Carbonic anhydrase 11	3.39 ± 0.35	3.74 ± 0.13	1.09	0.63
Car12	Carbonic anhydrase 12	0.01 ± 0.01	0.02 ± 0.01	not exp	n/a
Car13	Carbonic anhydrase 13	0.63 ± 0.11	0.64 ± 0.08	1.02	0.95
Car14	Carbonic anhydrase 14	75.2 ± 3.0	85.7 ± 3.7	1.13	0.02
Car15	Carbonic anhydrase 15	1.19 ± 0.16	1.39 ± 0.13	1.15	0.59

mRNA expression levels (RPKM values) of all carbonic anhydrase isoforms in WT and AE3-null (KO) heart are shown, with fold-change and P-values (n/a = not applicable). Car1, Car5a, Car6, Car10, and Car12 had no transcripts in one or more samples of both WT and KO samples (n = 4 of each genotype), and are therefore considered to be not expressed (not exp) in FVB/N mouse heart. Car14 facilitates CO_2 venting from mitochondria[14] and associates with AE3 on the extracellular surface of the myocyte[16] and other cell-types[13], was expressed at much higher levels than other isoforms, and was one of only two CAs (CA14 and CA5b) exhibiting significant expression changes (see text). Extracellular Car4, which also affects AE3 activity[77], was expressed at moderate levels in mouse heart and was not changed. These data support the predominance of Car14 in mouse cardiac myocytes. However, note that Car4 (CA IV) is the predominant isoform in human heart (see Supplementary Table 10).

Supplementary Table S9. Expression Levels (RPKM) of Cl^- Channel mRNAs in Heart.

Gene Symbol	Description	WT Mean ± SE	KO Mean ± SE	Fold Change	P-value
Clcn1	Chloride channel 1	1.94 ± 0.27	2.60 ± 0.17	1.33	0.04
Clcn2	Chloride channel 2	1.55 ± 0.07	1.69 ± 0.12	1.08	0.29
Clcn3	Chloride channel 3	10.6 ± 0.4	11.2 ± 0.4	1.04	0.41
Clcn4-2	Chloride channel 4-2	22.8 ± 0.4	23.8 ± 0.4	1.03	0.39
Clcn5	Chloride channel 5	0.60 ± 0.04	0.66 ± 0.05	1.09	0.53
Clcn6	Chloride channel 6	4.36 ± 0.36	4.45 ± 0.14	1.01	1
Clcn7	Chloride channel 7	11.7 ± 0.09	11.8 ± 0.30	1.00	1
Clcnkb	Chloride channel Kb	0.40 ± 0.10	0.45 ± 0.05	1.11	0.50
Ostm1	Osteopetrosis associated transmembrane protein 1	13.7 ± 0.3	11.18 ± 0.15	0.8	0.000023
Cftr	Cystic fibrosis transmembrane conductance regulator	0.07 ± 0.02	0.11 ± 0.02	1.59	0.31
Best 1	Bestrophin 1	0.07 ± 0.02	0.09 ± 0.02	1.32	0.63
Best 3	Bestrophin 3	5.07 ± 0.36	4.40 ± 0.06	0.86	0.04
Ano1	Anoctamin 1	4.72 ± 0.01	4.74 ± 0.11	1.00	0.96
Ano3	Anoctamin 3	0.11 ± 0.02	0.14 ± 0.01	1.28	0.55
Ano4	Anoctamin 4	2.0 ± 0.11	2.64 ± 0.14	1.30	0.001
Ano5	Anoctamin 5	0.93 ± 0.12	0.47 ± 0.03	0.50	0.001
Ano6	Anoctamin 6	8.43 ± 0.19	8.63 ± 0.2	1.01	0.95
Ano8	Anoctamin 8	8.51 ± 0.39	9.78 ± 0.34	1.13	0.01
Ano10	Anoctamin 10	25.8 ± 0.4	24.7 ± 0.97	0.94	0.25
Clca1	Ca^{2+} activated Cl^- channel 1	0.44 ± 0.03	0.39 ± 0.06	0.88	0.73
Clca2	Ca^{2+} activated Cl^- channel 2	0.15 ± 0.01	0.16 ± 0.02	1.05	0.90
Lrrc8a	Leucine rich repeat containing 8A	21.5 ± 1.61	20.0 ± 0.55	0.92	0.35
Lrrc8b	Leucine rich repeat containing 8B	3.00 ± 0.08	2.96 ± 0.15	0.97	0.75
Lrrc8c	Leucine rich repeat containing 8C	9.75 ± 0.33	8.57 ± 0.31	0.87	0.01
Lrrc8d	Leucine rich repeat containing 8D	1.69 ± 0.01	1.59 ± 0.06	0.93	0.37
Lrrc8e	Leucine rich repeat containing 8E	0.15 ± 0.03	0.20 ± 0.03	1.33	0.32

Expression levels and fold-changes of potential sarcolemmal/t-tubule Cl^- channels in WT and AE3-null (KO) heart. Genes not detected in one or more samples were considered not expressed (all had mean RPKM < 0.05) and are not included. The Clic (Chloride Intracellular Channel) isoforms also are not shown. The genes shown include those of the Clcn family[78]; Ostm1, an accessory subunit that interacts with Clcn7[78]; the Anoctamin and Bestrophin families, which function as Ca^{2+}-activated Cl^- channels[79,80]; Clca Ca^{2+}-activated Cl^- channels and Cftr, which were expressed at only low levels; and Lrrc8 isoforms, recently identified as components of the volume-regulated anion channel (VRAC)[81,82]. A clear pattern of changes indicative of Cl^- channel activity that might function in Cl^- recycling was not observed, although changes in Ostm1 and Lrrc8c transcripts suggest their involvement. Members of the Clcn family can function as either Cl^- channels (Clcn1, 2, and kb) or as Cl^-/H^+ exchangers (Clcn3, 4, 5, 6, and 7), most likely with a 2:1 stoichiometry[78]. Although the latter group typically function in endosomal compartments or lysosomes (Clcn7), Clcn7/Ostm1also functions in the ruffled border (a plasma membrane) of osteoclasts[78] and surface expression of members of this group has been reported. Their high expression suggests important functions in heart. If involved in Cl^- recycling to balance Cl^-/HCO_3^- exchange, which would be sharply reduced in the AE3 KO, then some of these changes could represent remodeling in direct response to reduced Cl^- uptake.

Supplementary Table S10. mRNA expression of H^+ extrusion mechanisms, Cl^-/HCO_3^- exchangers, and extracellular carbonic anhydrases in heart of mammalian species.

Gene	Protein	FVB/N Mouse[a]	Fisher Rat[b]	C57Bl6 Mouse[c]	Opossum[c]	Rhesus Monkey[c]	Human[c]
Slc9a1	NHE1	9.10 ± 0.20	2.4	8	7	17	5
Hvcn1	HVCN1	2.11 ± 0.13	1.1	1	1	5	9
Slc4a1	AE1	0.20 ± 0.05	3.1	0.2	2	0.5	0.3
Slc4a2	AE2	12.6 ± 0.28	3.6	7	16	23	7
Slc4a3	AE3	85.7 ± 0.64	25	47	45	77	178
Slc26a6	PAT1	4.56 ± 0.26	4	4	n/a	6	3
Car14	CA14	75.2 ± 3.0	24.9	41	101	3	1
Car4	CA4	10.6 ± 0.8	3.9	9	n/a	9	8

RPKM values for mRNA expression is from: [a]Current study, Mean ± SE for WT FVB/N mouse; [b]EBI Expression Atlas (see Supplemental Methods), mean values for Fisher 344 Rat[83], [c]EBI Expression Atlas, mean values for Mouse (C57Bl6), gray short-tailed opossum, rhesus monkey, and human[84]. Data were normalized in the latter study[84], allowing approximate cross-species comparisons of individual transporters; the other studies cannot be directly compared due to differences in the number of genes examined. These expression data indicate that HVCN1 is expressed in heart of all species listed and that AE3 is the most abundantly expressed Cl^-/HCO_3^- exchanger. Car14 is the most abundantly expressed carbonic anhydrase mRNA in rodent and opossum heart; Car4 is most abundant in human and rhesus monkey heart. Both CA isoforms enhance AE3 activity in neurons[77].
